# Wearable Sensors Fabricated by 3D‐Printed Composite Hydrogel with 2D Fillers

**DOI:** 10.1002/smtd.202502195

**Published:** 2026-01-30

**Authors:** Yaxuan Li, Sheng Pei, Jun Wang, Chuhan Zhang, Beichao Shi, Zhengtang Luo

**Affiliations:** ^1^ Department of Chemical and Biological Engineering Guangdong‐Hong Kong‐Macao Joint Laboratory for Intelligent Micro‐Nano Optoelectronic Technology William Mong Institute of Nano Science and Technology and Hong Kong Branch of Chinese National Engineering Research Center for Tissue Restoration and Reconstruction The Hong Kong University of Science and Technology Kowloon Hong Kong P. R. China; ^2^ Key Laboratory of Mechanism Theory and Equipment Design of Ministry of Education School of Mechanical Engineering Tianjin University Tianjin China

**Keywords:** 3D printing, conductive material, flexible sensor, hydrogel, microstructure

## Abstract

Flexible sensors demonstrate exceptional adaptability across human‐computer interaction, health monitoring, and robotic systems. However, sensing materials suffer from inadequate conformation capability and microstructural inaccuracies, resulting in function deficiencies. This review examines composite hydrogel formulations that incorporate conductive nanofillers, with particular emphasis on 2D nanomaterials, whose functional tunability enables precise regulation of electrical and interfacial properties. The strategic integration of microstructures further improves sensor sensitivity, durability, and environmental adaptability. We also examine implementation of flexible sensors based on 3D‐printed hydrogel in emerging applications including pH monitoring, glucose detection, and food safety assessment. We suggest that future development prioritize elucidating sensing mechanisms, achieving multifunctional integration, advancing material engineering, and refining precision manufacturing. Particularly promising research directions include developing intelligent tactile feedback systems for humanoid robots and creating capsule robot‐integrated platforms for gastrointestinal disease monitoring.

## Graphical abstract

This review explores how 3D printing integrates 2D conductive fillers into hydrogel matrices to fabricate high‐performance flexible sensors. By tailoring microstructures and nanomaterial interactions, these devices achieve enhanced sensitivity, durability, and environmental adaptability for healthcare monitoring, human‐machine interfaces, and robotic sensing applications.

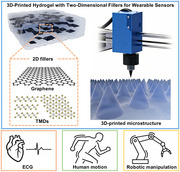

## 1 Introduction

Flexible sensors enhance monitoring capacities and allow the transduction of diverse stimuli including mechanical deformation, biochemical variations and environmental changes into quantifiable electrical signals. Unlike the rigid counterparts that suffer from limited adaptability in complex environments, the flexible sensors exhibit outstanding mechanical compliance, biological compatibility and conformal integration capabilities. Such characteristics have propelled the applications of flexible sensors in the fields of continuous health monitoring [1, 2, 3], adaptive perception in soft robotics [4, 5], remote and biomimetic control of industrial robot [2], [6] and intelligent food quality surveillance [7, 8].

The application scope of hydrogels in soft electronics has rapidly expanded, with notable progress in several key areas. This includes the development of extreme hydrogel bioelectronics engineered to maintain sensing and energy storage functions under harsh environmental conditions, such as extreme temperatures and mechanical stresses [9]. For instance, repetitive motion can lead to fatigue cracking, sweat or bodily fluids can cause swelling or detachment, and low‐temperature environments can freeze the hydrogel, degrading its conductivity and flexibility. To address these limitations, the emerging field of extreme hydrogel bioelectronics has developed advanced material strategies to endow hydrogels with enhanced tolerance against such harsh conditions. These include designing anti‐fatigue networks through energy‐dissipation mechanisms [10], implementing anti‐swelling and anti‐freezing chemistries [11, 12], and engineering robust and biocompatible interfaces for stable skin adhesion [13, 14]. By integrating these extreme properties, hydrogel‐based wearable sensors can maintain high performance and durability under real‐world operating conditions, enabling reliable monitoring of physiological signals, human motion, and biochemical markers even in demanding environments.

Significant efforts have also been devoted to creating skin‐like hydrogel‐elastomer hybrid devices, which utilize thin, porous polymer films as an intermediate layer to achieve robust yet comfortable skin contact for high‐fidelity, long‐term monitoring of biomarkers in biofluids like sweat [15]. One approach involves embedding an ultrathin layer of thermoplastic polyurethane (TPU) between the conductive component and the hydrogel. This interfacial structure not only prevents delamination between layers—even under tensile stress—but also brings the overall modulus close to skin‐like levels. Devices fabricated with this structure maintain comfortable and stable skin adhesion during movement, enabling reliable monitoring of sweat constituents such as pH, sodium ions, and potassium ions. Their sensing performance approaches ideal electrochemical behavior, with repeatable readings even under moderate strain conditions. Another study [16] in this field utilized Ti_3_C_2_T*_x_* MXene nanosheets to fabricate flexible electrodes featuring both high transparency (60%@550 nm) and low sheet resistance (200 Ω/sq), which were further assembled into capacitive tactile sensors. The device maintains stable photoconductive performance after 1000 bending cycles and accurately recognizes multiple interactive gestures including proximity, touch, and pressure, demonstrating the potential of 2D materials for constructing high‐resolution, environmentally stable transparent sensing interfaces. To further enhance skin‐friendliness and user experience during prolonged wear, a study [17] proposed an “in situ growth” strategy. By integrating PAM‐LiCl hydrogels with silk‐based knitted fabrics, researchers developed an ion‐electronic touch panel with a textile‐like tactile feel. This device not only exhibits significantly enhanced mechanical strength (114 MPa) but also demonstrates excellent breathability and biocompatibility. It maintains ultrahigh tactile resolution under deformations, such as stretching and bending, enabling advanced human–machine interaction functions like virtual reality handwriting interaction and gesture recognition.

In recent years, the development of multifunctional hydrogels has become a hot topic, aiming to integrate multiple functions such as sensing, actuation, therapy, self‐healing, and environmental responsiveness within a single material system to construct intelligent, adaptive integrated systems. The core of multifunctional hydrogels lies in functional integration and synergy. For instance, in intelligent wound management, hydrogel dressing can simultaneously possess antibacterial, hemostatic, and self‐adhesive biological functions while integrating pH sensing capabilities to monitor wound infection status in real time [18]. By combining 3D printing technology, the dressing achieves precise geometric matching with the wound. Analyzing sensor signals through machine learning algorithms enables a closed‐loop intelligent management system of “monitoring‐assessment‐treatment.” This transcends the passive covering role of traditional dressings, demonstrating the immense potential of hydrogels as active smart medical platforms. Furthermore, by incorporating dynamic covalent bonds [19], multiple noncovalent interactions, or nanocomposites, hydrogels gain additional properties such as self‐healing, high stretchability, freezing resistance, and conductivity. This enables them to withstand complex mechanical deformations and extreme environments while reliably functioning in human‐machine interaction scenarios like wearable sensing and soft robotics. Multifunctional hydrogel sensors provide a flexible foundation for applications that require several sensing functions to operate together, such as human‐machine interaction or soft robotic feedback systems.

The performance of flexible sensors is fundamentally governed by the intrinsic properties of the sensitive layer materials, which comprise of conductive nanofillers and polymeric matrices. This composite combines the electrical conductivity imparted by nanoscale additives such as metallic nanoparticles [20], nanowires [21], and conjugated polymers [22] with the mechanical compliance and biocompatibility provided by elastomeric substrates including polydimethylsiloxane (PDMS) [23], Ecoflex [24], polyurethane [25], polyimide [26], and hydrogels [27]. Compared with other polymeric matrices, hydrogels emerge as particularly promising matrix materials due to their unique 3D porous networks that exhibit exceptional mechanical deformability, softness and stimuli‐responsive characteristics to environmental variations including mechanical stress, temperature and ionic concentration changes. The application scope of hydrogels in soft electronics has rapidly expanded, with significant progress in several key areas. This includes the development of adaptable conductive hydrogel‐enabled soft electronics, which are engineered to overcome challenges like icing, water loss, and mechanical damage in extreme environments, thereby ensuring reliable performance under harsh conditions such as extreme temperatures and physical deformation [28]. Concurrently, conductive hydrogel‐enabled electrodes have emerged as a pivotal solution for scalp electroencephalography (EEG) monitoring, effectively overcoming the compatibility barriers posed by the hairy scalp interface to achieve high‐quality, long‐term neural signal acquisition [29]. Furthermore, advances in ionic conductive hydrogels for skin sensor applications have yielded materials with excellent biocompatibility, flexibility, and the ability to mimic biological ion transport, making them ideal for long‐term, comfortable health monitoring [30]. These material‐level innovations, focused on enhancing environmental adaptability, bio‐integration, and sensing fidelity, provide a solid foundation for their integration with advanced manufacturing techniques like 3D printing to achieve superior structural and functional control.

The incorporation of microstructures onto the sensitive layer of flexible sensors has become a significant approach to enhance electromechanical performance, such as the sensitivity and dynamic range [31, 32, 33]. Such microstructures improve signal transduction efficiency by generating geometrically amplified contact area variations when external stimuli are applied, producing more pronounced resistance or capacitance changes while maintaining structural integrity during cyclic deformation. Conventional fabrication techniques such as photolithography [34], spray coating [35], and sputtering [36] have demonstrated feasibility in creating such microstructured surfaces. However, their practical implementation is frequently constrained by multistep processing requirements, compromised scalability and limited design flexibility. Recently, the development of 3D printing technologies has revolutionized this fabrication process. By adopting layer by layer manufacturing method, 3D printing is used to fabricate microstructures at multiple scales and ensure the effective interfaces among. This fabrication methodology not only overcomes the geometric constraints of traditional subtractive processes but also provides possibilities for creating sensors with tailored electromechanical responses.

**FIGURE 1:**
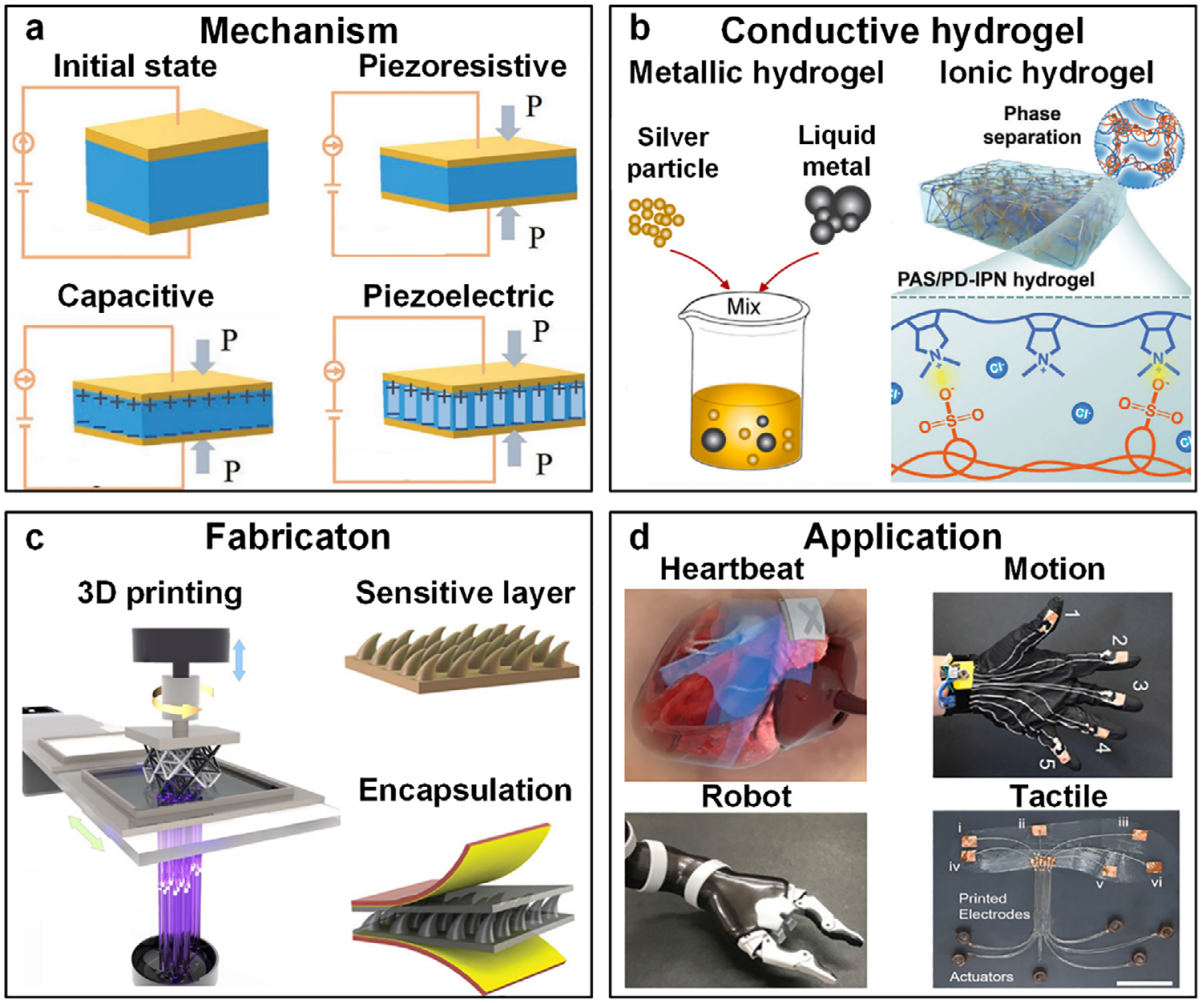
Schematic overview of hydrogel‐based flexible sensors fabricated via 3D printing technology. (a) Working mechanism of the wearable sensors. Reproduced with permission [37]. Copyright 2021, Wiley‐VCH GmbH. (b) The conductive hydrogel used to fabricate the wearable sensor. Reproduced with permission [38]. Copyright 2025, Elsevier Ltd. Reproduced with permission [39]. Copyright 2024, Wiley‐VCH GmbH. (c) The fabricating process of wearable sensor via 3D printing. Reproduced under the terms of the CC‐BY license [40]. Copyright 2023, Springer Nature. Reproduced under the terms of the CC‐BY license [41]. Copyright 2023, Wiley‐VCH GmbH. (d) The applications of the wearable sensors. Reproduced under the terms of the CC‐BY license [42]. Copyright 2023, Springer Nature. Reproduced with permission [43]. Copyright 2021, Wiley‐VCH GmbH. Reproduced with permission [44]. Copyright 2025, Elsevier B.V.

This review is motivated by the need to address a persistent challenge in soft sensor development: integrating high electrical conductivity into hydrogel‐based devices without compromising their mechanical compliance or manufacturability. While hydrogels offer exceptional softness and biocompatibility, and 3D printing enables architectural control, achieving robust electronic functionality has traditionally required high loadings of conductive fillers that degrade mechanical properties and hinder printability. The emergence of 2D conductive nanomaterials—notably MXenes, graphene derivatives, and transition metal dichalcogenides—provides a critical solution. Their high aspect ratio and specific surface area allow the formation of conductive networks at very low volume fractions, thereby preserving the hydrogel's intrinsic stretchability and compatibility with advanced printing processes. Unlike broader reviews that catalog hydrogel sensors or 3D‐printed wearables in general [37, 45, 46, 47, 48], this article offers a focused examination of the synergistic triad of 3D printing, hydrogel matrices, and 2D conductive fillers. We systematically dissect how printing parameters govern the integration of nanofillers and the fabrication of functional microstructures, and how these structural features in turn determine sensor performance. In doing so, we move beyond a summary of existing devices to establish process‐structure–property relationships that can guide rational sensor design. Concurrently, 3D printing technology offers capabilities in customized fabrication, enabling precise control over sensor architecture and functionality. The 3D‐printed hydrogels present transformative opportunities for developing personalized sensors with high performance. As illustrated in Figure 1, we summarize the latest state‐of‐the‐art in 3D‐printed hydrogel‐based sensors. First, the potential of 3D printing technologies used to fabricate hydrogel‐based sensor is discussed. Subsequently, we analyze the nanoscale conductive fillers, which impart electrical properties. Moreover, the performance improvement of hydrogel‐based sensors by adopting microstructures is highlighted. Then, the discussion expands to applications, including human–machine interface, food safety detection and robotic manipulation. Finally, we present forward‐looking perspectives on 3D‐printed hydrogel sensors.

## 2 3D Printing Technologies Using Hydrogel‐Based Sensor Fabrication

3D printing originated in 1980s with the advancement of stereolithography (SLA), which utilizes ultraviolet (UV) lasers to cure liquid photopolymer resin layer by layer [49]. In the early 2010s, hydrogels were introduced into the 3D printing process, enabling the fabrication of complex hydrogel‐based structures [50]. With the development of 3D printing technologies, the manufacturing precision has been improved, thereby advancing the development of flexible sensors. Notably, 3D printing offers significant advantages in flexible sensors fabrication, including customization capabilities, design flexibility and reduced material waste, while simplifying the fabrication of intricate geometries.

3D printing can be divided into various techniques based on working principles, including material extrusion, vat photopolymerization, powder bed fusion, and material jetting. However, due to the unique rheological properties and functional requirements of conductive hydrogels, not all 3D printing methods are suitable for fabricating hydrogel‐based flexible sensors. Current research indicates that digital light processing (DLP), SLA and direct ink writing (DIW), have emerged as the predominant 3D printing strategies for manufacturing hydrogel‐based flexible sensors. These methods enable precise control over hydrogel network formation through photoinitiated polymerization and shear‐thinning fluid deposition, offering advantages in material compatibility, structural resolution, and fabrication flexibility. The subsection provides a comprehensive overview of these prevalent fabrication methodologies.

### 2.1 Digital Light Processing

The main components of ‌DLP system include light source, build platform and cure source [51]. DLP 3D printing method fabricates devices by layer‐wise photopolymerization of conductive/elastomeric photoresins under UV light, which fabricate the microstructures with precision of 50–100 µm. Hydrogels suitable for DLP 3D printing require high photoreactivity and rapid curing kinetics, typically formulated as low‐viscosity liquid precursors (10–500 mPa s) to ensure fast resin recoating and high‐resolution layer‐by‐layer fabrication. The resin must contain efficient photoinitiators and polymerizable groups (e.g., acrylate or methacrylate) that respond to specific wavelength light, enabling precise and uniform cross‐linking within short exposure times. Representative systems include chemically modified natural polymers (e.g., gelatin‐methacryloyl, hyaluronic acid methacrylate) and synthetic photopolymers (e.g., poly ethylene glycol diacrylate), with optimized optical properties and layer adhesion. This technology has been leveraged to develop advanced hydrogel‐based flexible sensors, benefiting from its rapid manufacturing and microstructural control.

DLP 3D printing is categorized into bottom‐up and top‐down approaches based on the relative position between light source and build platform [52]. The bottom‐up approach refers to a layer‐by‐layer photopolymerization process where UV light is projected through a transparent build platform located beneath the resin vat, enabling precise curing of photopolymer resin in direct contact with the optical window, thereby achieving high‐resolution fabrication with minimized light distortion. However, this method imposes constraints on build volume and requires careful optimization of resin viscosity and antiadhesion coatings to mitigate interfacial delamination forces during layer separation [53]. In top‐down DLP 3D printing, the photopolymerization process initiates at the air‐resin interface with the light source positioned above the resin vat. The build platform progressively descends into the liquid photopolymer reservoir, while each newly cured layer remains adhered to the preceding layer, this configuration enables larger build volumes by eliminating constraints imposed by transparent vat bottoms. Nevertheless, it is necessary to achieve precise control of resin meniscus stability and oxygen inhibition effects to maintain dimensional accuracy [40]. Additionally, the higher separation forces during layer detachment provides the requirement of optimizing retraction velocity and silicone‐based release coatings to reduce interfacial failures.

**FIGURE 2:**
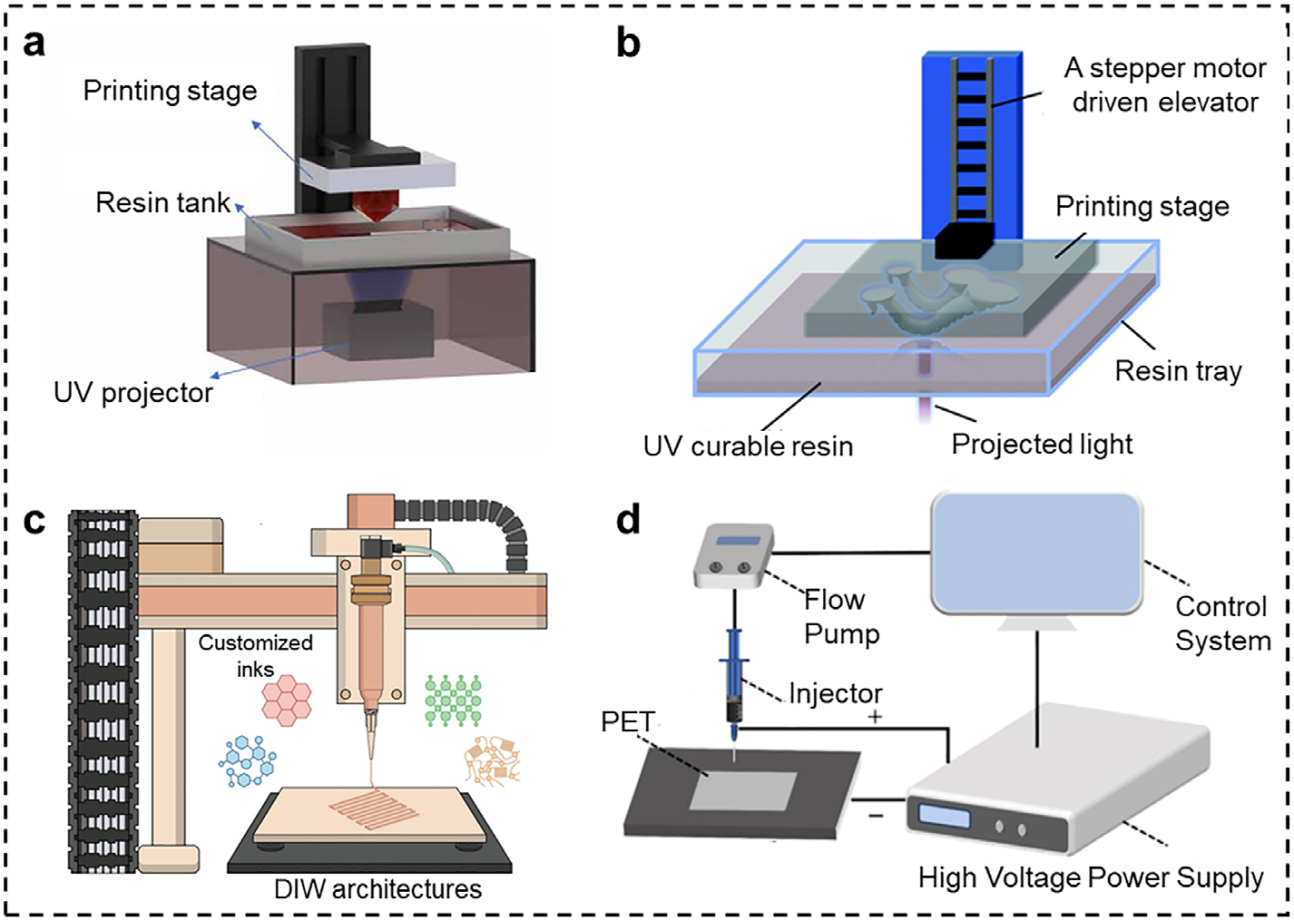
3D‐Printing functional hydrogels. (a) Diagram of the Digital Light Processing (DLP) 3D printing system with a UV projector. By printing layer by layer, the designed 3D model will be manufactured. Reproduced with permission [59]. Copyright 2021, IOP Publishing Ltd. (b) Schematic of projection stereolithography 3D printing, which selectively create the desired structure of near arbitrary geometry with micrometer‐scale resolution due to its point‐by‐point laser scanning mechanism. Reproduced with permission [65]. Copyright 2019, Royal Society of Chemistry. (c) Schematic illustration of the Direct Ink Writing (DIW) 3D printing, achieving a layer‐by‐layer fabrication of 3D structures using ink dispensed from a micro‐nozzle. Reproduced with permission [67]. Copyright 2023, Elsevier Ltd. (d) Schematic representation of the electrohydrodynamic (EHD) printing process, where the hydrogel precursor forms a jet at the top of the nozzle under the influence of a high‐voltage electric field. Reproduced with permission [75]. Copyright 2024, Elsevier B.V.

The practical application of hydrogel‐based flexible sensors is significantly hindered by two critical challenges: dehydration‐induced shrinkage when exposed to ambient conditions and signal drift caused by morphological changes in sensitive layers during prolonged operation. To address these limitations, voxel‐level precise arrangement of heterogeneous materials through dual‐material 3D‐printing has emerged as a promising solution, which combines complementary material properties while maintaining structural integrity [54]. In this approach, an ionic hydrogel (e.g., PAAm/PEGDA/Mg^2+^) is alternately printed with a water‐dilutable polyurethane acrylate (WPUA) layer. The two photocurable resins share compatible acrylic chemistry, allowing them to form a covalent bond at the interface during UV curing, resulting in a monolithic, multilayer structure. The WPUA acts as a hydrophobic barrier that significantly reduces water vapor transmission, thereby mitigating dehydration‐induced shrinkage and resistance drift. Meanwhile, the chemically bonded interface eliminates relative slippage between layers under cyclic loading, ensuring stable signal output. For instance, a reported five‐layer ionic skin fabricated by this method retained over 96% of its initial mass after 7 days of ambient exposure and showed less than 5% capacitance drift after 10 000 compression cycles, demonstrating superior long‐term performance stability [54]. Considering the principle of DLP method, it is a key step to optimize photoinitiator concentrations in each precursor resin to achieve matched curing kinetic and ensure mechanical compatibility between dissimilar photopolymers. In addition, material cross‐pollution poses a challenge in multimaterial DLP 3D printing. To solve this problem, various clearing approaches have been reported, including mechanical wiping [55], fluid jetting [56], ultrasonic cleaning [57], and centrifugal purification [58]. For example, ionic skins were fabricated through alternating printing of two photocurable materials‐hydrogel and water‐dilutable polyurethane acrylate (WPUA)‐using DLP 3D printing strategy, as shown in Figure 2a [59]. And the ultrasonic vibration method was adopted to clear the prepolymer during the manufacturing process of adjacent layers to avoid material pollution. The centrifugal DLP multimaterial 3D printing system enables fabrication of large‐scale complex heterogeneous architectures, spanning hydrogels, soft/rigid polymers, shape‐memory polymers, conductive elastomers and even ceramics. Inspired by mammalian centrifugal dehydration mechanisms, the platform implements rapid rotational motion during material switching to achieve residue‐free material transition through centrifugal purification. This contactless purification approach demonstrates remarkable insensitivity to structural dimensions, geometric complexity and resin viscosity [40].

### 2.2 Stereolithography

SLA as an alternative implementation of vat photopolymerization‐based 3D printing technology, employs a focused laser beam to selectively cure photopolymer resin in a point‐by‐point scanning manner [60]. This technique enables the fabrication of complex 3D architectures with high geometric freedom, which are challenging to achieve through conventional manufacturing methods. The accuracy of SLA‐printed components is directly determined by the laser system's positioning precision and the printing resolution can reach sub‐100 nm [61, 62]. Hydrogels compatible with SLA 3D printing are formulated as photo‐curable precursors with tailored rheological properties. A typically low viscosity range (1–5 Pa s) is essential to facilitate rapid and uniform resin recoating between layers, ensuring printing precision. The ink must incorporate effective photoinitiators and polymerizable functional groups (e.g., acrylate or methacrylate) to enable controlled, rapid cross‐linking under specific wavelength exposure, which is critical for achieving high‐resolution fabrication and excellent shape fidelity. Commonly employed systems include chemically modified natural polymers (such as gelatin‐methacryloyl) and synthetic photopolymers (e.g., poly ethylene glycol diacrylate). Projection SLA (P‐SLA) 3D printing has been applied for fabricating highly conductive and stretchable hydrogel‐based flexible sensors [63]. The P‐SLA technique enabled high‐resolution patterning of silver flake‐embedded alginate‐polyacrylamide hydrogels, creating customized wireless eye movement tags capable of stable operation with precise motion detection and fast response. SLA 3D printing strategy was adopted to fabricate hydrogel‐based pH sensors, where the thickness of each layer was only 50 µm. The pH sensor demonstrated linear response across the physiological range of 2–7 [64].

Compared to DLP method, SLA 3D printing strategy exhibits inferior printing efficiency and higher operational costs due to its point‐by‐point laser scanning mechanism. Since the polymerization quality depends critically on energy control, parameters such as laser power intensity, exposure duration and scanning velocity must be optimized to achieve the desired curing depth and dimensional accuracy, which are essential for high‐resolution fabrication. Previous researchers reported an improved SLA platform (Figure 2b) which integrates zwitterionic chemistry and a simple but efficient aqueous photoinitiation system [65]. One zwitterionic solution with medium crosslink density was selected to demonstrate the capability of the SLA compatible Z‐gels to print soft octopus arms. The 3D fabrication of this hydrogel was performed at a rate of 14s per layer at a high resolution (50 µm thickness each layer) through adjusting the energy dosage. A multimaterial 2D hydrogel patterning was achieved by integrating a customized miniature pump into an SLA 3D printer [66].

### 2.3 Direct Ink Writing

Shear‐thinning behavior and yield stress properties are the essential characteristics of the inks used in DIW, where the inks are extruded out under external pressure. During operation, these specially engineered inks transition from liquid‐like flow under shear stress during extrusion to rapid solidification upon deposition, enabling the fabrication of complex 3D architectures with structural integrity. The inherent shear‐induced extrusion mechanism (Figure 2c) of DIW fundamentally enables its exceptional material processing capability, which spans a broad spectrum of functional materials including conductive polymers, ceramic‐polymer composites and biologically active components [67]. This unique characteristic positions DIW as a particularly suitable platform for fabricating hydrogel‐based architectures, where precise spatial control of soft hydrated networks is essential for maintaining structural integrity and bio‐functionality. And the excellent resolution is maintained through adjustable parameters like nozzle diameter, printing speed and extrusion pressure. To achieve high‐fidelity printing with superior pattern resolution and structural integrity, one has to optimize the key 3D printing parameters, including nozzle diameter, deposition velocity, and extrusion pressure [68]. Hydrogels compatible with direct ink writing (DIW) 3D printing primarily encompass natural polymer‐based systems (e.g., alginate, gelatin, chitosan), synthetic polymers (e.g., polyethylene glycol derivatives, thermosensitive Pluronic F127), and composite or functionalized formulations (such as nanoclay‐reinforced hydrogels, double‐network hydrogels, and cell‐laden bioinks). While DIW conventionally relies on bioinks with viscosities of 10^−1^–10^3^ Pa s to ensure structural fidelity, this requirement typically precludes the use of low‐viscosity hydrogel precursors (∼10^−3^ Pa s). However, recent advances in interfacial engineering enable the successful printing of such dilute inks via DIW, broadening its material versatility. By precise tailoring the composite ink formulation to modulate its rheological behavior, an optimal balance between the material properties and processing parameters was established. When fabricated into strain sensors, the resulting devices demonstrated remarkable operational stability, maintaining consistent performance over 1000 loading cycles across varying frequency regimes. Different from curing at room temperature, some groups [69] developed a hybrid printing method, where the shear‐thinning hydrogel precursor is extruded through a nozzle to form microfilaments, with subsequent UV irradiation significantly accelerating the curing speed to enhance printing efficiency. Additionally, an innovative approach has been developed by combining DIW 3D printing with freeze‐drying methods, which enabled effective reorientation of nanofillers and removal of hydrogels [70]. The time cost of production was significantly reduced. Notably, the 6 mm‐thick sensors prepared via the DIW‐freeze drying approach demonstrated substantially enhanced sensitivity.

Material deposition, typically achieved through physical additive processes, enables facile integration of diverse materials. The incorporation of multinozzle configurations further facilitates multimaterial printing, offering particular advantages for the development of flexible sensors. This approach synergistically combines material flexibility, resolution control, cost‐effectiveness, and rapid prototyping capabilities, making it highly suitable for advanced sensor fabrication. A dual‐nozzle DIW system was demonstrated by employing hydrogel inks with distinct rheological and electrical properties to fabricate microdome structures of varying dimensions on sensor sensitive layers [71]. Another work addressed the patterning challenge of chromogenic structures by developing a multimaterial microgel DIW system featuring integrated multinozzle deposition [72]. The approach combined UV irradiation to fabricate mechanically responsive double‐network (DN) hydrogels that exhibited characteristic mechanochromic behavior under tensile or compressive strains. Instead of integration of multinozzle, an innovative single‐nozzle DIW system employing four material reservoirs with programmable deposition sequences was utilized [73]. Similarly, UV‐assisted curing was employed to enhance printing efficiency while maintaining excellent biocompatibility in the fabricated products.

### 2.4 Electrohydrodynamic

Electrohydrodynamic (EHD) 3D printing utilizes electric‐field‐induced fluid jetting to deposit functional materials with micro/nanoscale precision. Hydrogel precursors for EHD 3D printing must exhibit low viscosity, typically between 0.001 and 0.1 Pa s. The hydrogel should possess a relatively high dielectric constant, preferably in the range of 40–80, to enhance polarization and electric‐field manipulation. Meanwhile, surface tension should be tuned below 50 mN/m to reduce the threshold voltage for jet initiation and improve printing stability. Typical hydrogel suitable for EHD printing include low‐concentration sodium alginate, polyvinyl alcohol (PVA) solutions, and functionally modified conductive or composite inks. This method offers distinct advantages, including high‐resolution patterning, compatibility with diverse materials (polymers, ceramics, and composites) and low‐temperature processing suitable for flexible substrates. However, it also faces challenges such as complex parameter optimization, limited throughput due to single‐nozzle configurations and stringent requirements for ink properties, such as electrical conductivity and viscosity.

Recent studies have demonstrated an innovative strategy for fabricating high‐performance hydrogel‐based flexible sensors by integrating MXene nanosheets into hydrogel matrices via EHD printing (Figure 2d) [74, 75, 76]. Under applied electric fields, the MXene nanosheets exhibit electrophoretic alignment, facilitating their directional migration and subsequent formation of an interpenetrating network with the hydrogel polymer chains. This electric‐field‐assisted assembly process promotes the development of a more uniform and densely packed porous conductive network within the hydrogel matrix, which significantly enhances both mechanical properties and sensing capabilities. Additionally, a high‐performance ionic gel‐based flexible strain sensor is developed through a combined EHD printing and UV crosslinking approach [77]. EHD printing was employed to precisely deposit the ionic gel precursor within a PDMS mold, followed by UV‐induced photopolymerization to construct a robust double network (DN) structure. The resulting ionic gel membrane exhibited an optimal ionic conductivity of 1.231 × 10^−3^ S/cm and a rapid response time of 81 ms, while maintaining excellent flexibility and durability. This innovative fabrication method enabled the production of highly sensitive strain sensors capable of accurately detecting various human motions. These studies highlight the effectiveness of EHD printing for developing advanced conductive hydrogel with tailored electromechanical properties.

A major difficulty met by hydrogel‐based 3D‐printing techniques is that the conditions needed for smooth printing rarely align with the conditions required for robust, high‐performance soft sensors. In DLP and SLA, the precursor solution must be reasonably transparent and remain in a low‐viscosity range (roughly 0.01–5 Pa s) so that light can penetrate 200–300 µm into the resin and cure each layer uniformly. In practice, the introduction of even a small number of functional additives tends to scatter light and reduce the curing depth to well below 80 µm. This will lead to insufficient crosslinking and weak bonding at the interfaces of the layer. DIW operates under the opposite constraint. It tolerates much higher viscosities (10^1^–10^3^ Pa s) and supports a wider variety of hydrogel formulations, but the printable window is narrow. An increase in solid content commonly raises the yield stress dramatically, which in turn causes nozzle clogging, filament deformation after deposition, and slow viscoelastic recovery. When multiple inks are printed together, differences in their storage moduli often result in poor interfacial adhesion or tearing during deformation. EHD printing can reach sub‐micrometer resolution, but only when the ink falls within a very strict range of conductivity (around 10^−6^–10^−4^ S/m) and viscosity (10^−3^–10^−2^ Pa s). Many hydrogel precursors are out of this range, leading to frequent satellite droplets or interrupted jetting. These limitations make it clear that no single printing technique can simultaneously meet the demands of high resolution, broad materials compatibility, and mechanical reliability. Table 1 provides an overview of the characteristic requirements of each method and illustrates why their capabilities only partially overlap. A promising direction is the use of hybrid manufacturing approaches, for example, combining DIW's strength in multimaterial deposition with the fine patterning achievable by DLP. Such strategies allow structures and functions to be assigned to different stages of the printing process rather than forced into a single technique. Developing these process‐structure–property relationships will be essential for making hydrogel‐based printed sensors more reproducible and scalable.

**TABLE 1:** Summary of 3D printing techniques for hydrogel sensors.

Technique	Key requirements for hydrogel formulations	Suitable hydrogel types	Resolution & advantages	Limitations
DLP	Photocurable resins; viscosity from 0.01 to 0.5 Pa s; photoinitiator concentration 0.1–1 wt%.	Natural polymers (e.g., gelatin‐methacryloyl, hyaluronic acid methacrylate), and synthetic photopolymers (e.g., poly ethylene glycol diacrylate)	50–100 µm; fast printing.	Limited by vat size; oxygen inhibition.
SLA	Photocurable resins; viscosity 1–5 Pa s; laser power 10–100 mW.	Natural polymers (such as gelatin‐methacryloyl) and synthetic photopolymers (e.g., poly ethylene glycol diacrylate)	Sub‐100 nm; high precision.	Slower than DLP; higher cost.
DIW	Shear‐thinning inks with yield stress >100 Pa; viscosity 10^−1^–10^3^ Pa s.	Extrudable DN hydrogels (e.g., PVA/κ‐carrageenan); filler‐loaded inks.	100–500 µm; multinozzle.	Nozzle clogging with high fillers.
EHD	Electrically conductive inks; dielectric constant 40–80; viscosity 10^−4^‐0.1 Pa s; surface tension <50 mN/m.	Low‐concentration sodium alginate, polyvinyl alcohol (PVA) solutions, and functionally modified conductive or composite inks.	Micro/nanoscale; high precision.	Low throughput; parameter optimization.

## 3 Conductive Nanomaterials

**FIGURE 3:**
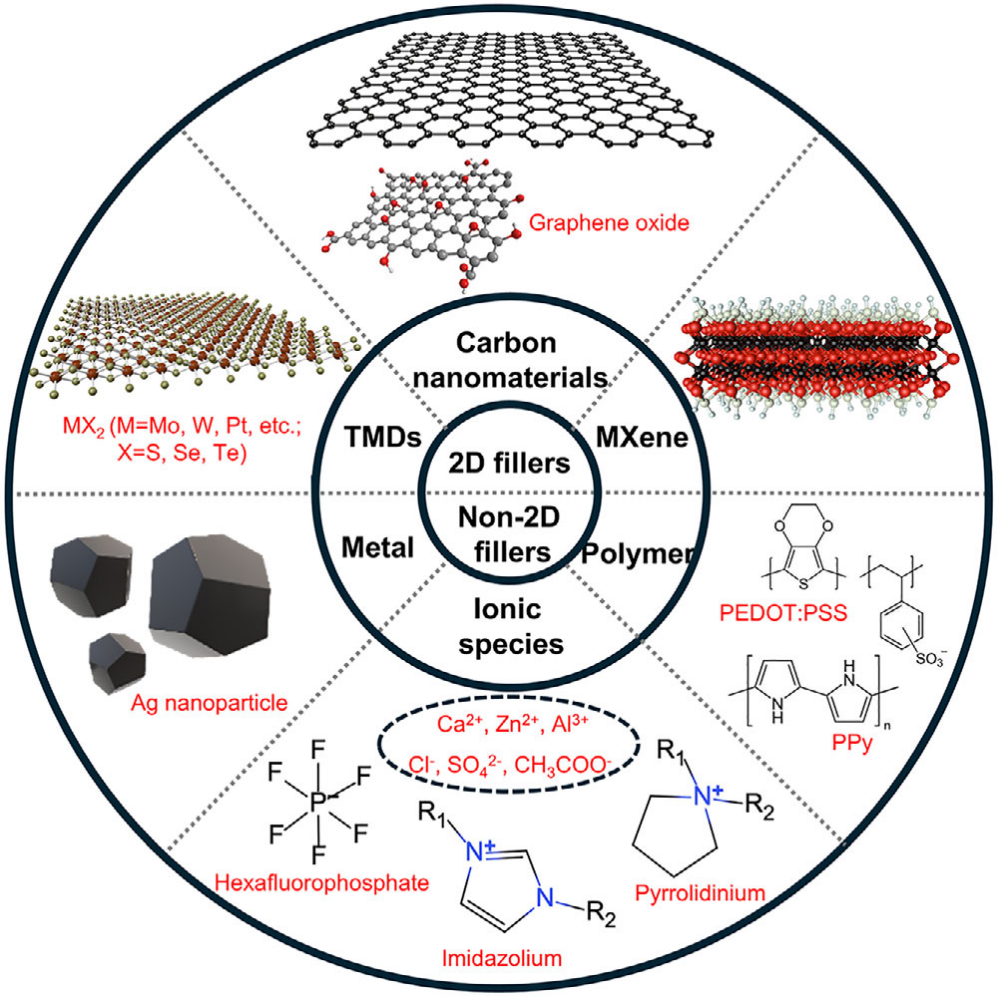
Conductive fillers for 3D printing. This illustration classifies nanofillers for 3D printed hydrogel sensors based on their structural dimensions. The 2D materials section features carbon nanomaterials, MXenes, and TMDs, emphasizing their layered structures and high surface‐area‐to‐volume ratios. The non‐2D materials category includes metallic nanomaterials, conductive polymers, and ionic species.

3D printed hydrogels for sensing applications typically comprise polymeric matrices and conductive nanofillers. The polymeric matrices provide essential mechanical stability, while the conductive nanofillers serve as functional elements, endowing the hydrogel with sensing capabilities. Among these, 2D nanomaterials—including graphene‐based materials, transition metal dichalcogenides (TMDs), and MXenes—have emerged as the most promising conductive fillers for 3D‐printed hydrogel sensors owing to their ultrahigh aspect ratios (>1000), atomically thin layered structure, abundant surface functional groups, and excellent solution processability (Figure 3). These intrinsic features enable dramatically lower percolation thresholds (typically 0.1–2 vol%, far below 5–20 vol% for 0D/1D fillers), superior interfacial compatibility with hydrogel networks, and shear/electric‐field‐induced alignment during DIW or EHD printing, resulting in highly anisotropic and robust conductive pathways even at ultralow loadings. Such advantages simultaneously deliver high electrical conductivity (>1000 S/m in many cases), large stretchability (>1000%), rapid response, and minimal mechanical reinforcement penalty, making 2D fillers uniquely suited for high‐performance, soft, and fully printable bioelectronics. Based on their structural dimensionality, the conductive nanofillers employed in these 3D printed hydrogels can be broadly categorized into the aforementioned 2D fillers along with non2D fillers, such as metallic nanomaterials, ionic species, and conductive polymers. Table 2 summarizes the performance of hydrogel‐based flexible sensor with different conductive nanomaterials fabricated by 3D printing and Figure 5 is plotted to compare the maximum GF and conductivity of different kinds of hydrogel‐based flexible sensors.

**TABLE 2:** Summary of hydrogel‐based flexible sensors fabricated by 3D printing.

Hydrogel materials	Printing method	Maximum GF (gauge factor)	Conductivity (Sm^−1^)	Stretchability	Refs.
PAM/CS/ PDADMAC	DLP	1.06	0.68	1473%	[105]
PAP/ ionic liquids/tannic acid	DLP	3	N/A	3500%	[106]
Polyacrylamide/ SPA	DLP	24.87	3.3	N/A	[107]
P(ACMO)/Pt	DLP	7.2	1.6	1781.5%	[108]
P (UA‐*co*‐AM‐*co*‐AA)/FeCl_3_	DLP	1.02	9.64	583%	[109]
ACMO/PEGDA/NaCl	SLA	3.2	2	3920%	[110]
Alginate‐Polyacrylamide/Ag	SLA	N/A	38 700	500%	[63]
AA/AETA /MBA/ SiO_2_‐SO_3_^−^ Na^+^	SLA	N/A	2.9	425%	[111]
VP‐SPB‐EG	DIW	27.82	0.086	1850%	[112]
PVA‐PAAm‐CaCl_2_	DIW	0.375	0.53	150%	[113]
PVA/PANI/CNC	DIW	4.1	3.35	550%	[114]
Ag/NPs/ CNTs/DS	DIW	78.6	N/A	463%	[115]
Ag flakes/silicone rubber	DIW	1.5	8500	150%	[116]
Amine‐PDMS/Ag	DIW	45.1	50	423.1%	[117]
PVA/Nanosilicate/Borax/NaCl	DIW	1.24	4.2	800%	[118]
MWCNT/CNCs	DIW	1.8	5	1000%	[119]
PAm/CS/CMC/CNT	DIW	6.859	N/A	1000%	[120]
PU/PVA/Ti_3_C_2_T*_x_*	DIW	5.7	72 570	424%	[92]
P407/QCS‐PAAm	DIW	0.5	0.29	1436.1%	[121]
PAM/CNF/MXene	EHD	6.73	2.7 × 10^−3^	550%	[76]
PVA/CMC‐Na/NaTFSI	EHD	2.09	34.27	338.19 %	[122]
P (AA‐*co*‐AM)/NaCl	EHD	1.74	0.2256	639.73%	[123]

### 3.1 Carbon Nanomaterials

Carbon nanomaterials, particularly carbon nanotubes (CNTs) and graphene oxide (GO), serve as predominant conductive nanofillers in 3D printed hydrogels. Their implementation is driven by three attributes: exceptional electrical conductivity, high specific surface area, and significant mechanical reinforcement capabilities, collectively enabling enhanced sensing performance in hydrogel‐based sensors.

CNTs are seamless cylinders formed from one or more concentric layers of graphene, classified as single‐walled nanotubes (SWNTs) or multiwalled nanotubes (MWNTs), and can have either open or closed ends [78]. Mechanically, SWNTs exhibit exceptional stiffness and their Young's modulus ranges from 0.96 × 10^12^ to 1.04 × 10^12^ Pa, varying inversely with nanotube diameter (approximately 4–35 Å) [79]. Electrically, pure CNTs demonstrate remarkably high conductivity, reaching values from 10^6^ to 10^7^ S/m. This places their conductivity on par with the best metallic conductors, silver (6.30 × 10^7^ S/m) and copper (5.96 × 10^7^ S/m) [80]. Complementarily, GO a 2D derivative of graphene‐provides distinct advantages in hydrogel integration: its residual oxygen‐containing functional groups enable superior aqueous dispersion, while the restored *sp* ^2^ network facilitates efficient conductive pathways within polymeric matrices, synergistically enhancing both electrical conductivity and mechanical flexibility of composite hydrogels [81].

The selection of these carbon nanomaterials is driven by their dual capacity to significantly enhance hydrogel electrical conductivity while simultaneously reinforcing mechanical properties, thereby enabling sensors with superior sensitivity and accelerated response times. Research has demonstrated the engineering of single‐walled carbon nanotube/poly(3,4‐ethylenedioxythiophene):poly(styrene sulfonate)‐F127 diacrylate hydrogels that achieve concurrent ultralow modulus (9 × 10^4^ Pa), extreme stretchability (520%), and high conductivity (440 S/m), facilitating multimodal epidermal sensing and sub‐1.5 V neurostimulation applications [82]. Beyond epidermal interfaces, a separate study developed a 3D printed mesenchymal stem cell‐laden graphene oxide‐fibrinogen patch that enhanced epicardial electrical integration via connexin 43 upregulation, while providing mechanical support and antiapoptotic protection to implanted cells, ultimately restoring cardiac function post myocardial infarction [83].

### 3.2 TMDs

TMDs represent a class of layered 2D materials with the general formula of MX_2_, where M denotes a transition metal (e.g., molybdenum or tungsten) and X represents a chalcogen element (e.g., sulfur, selenium, or tellurium). The electronic properties of TMDs are dictated by the combination of the transition metal d‐electron count and its coordination geometry [84]. For widely studied group 6 TMDs (e.g., MoS_2_, WS_2_), the 2H phase is semiconducting while the 1T/1T′ phase is metallic [85]. This tunability enables versatile functionality as nanofillers in hydrogels. For instance, electrodes printed via direct ink writing using highly concentrated 1T′/1T‐phase molybdenum disulfide/titanium disulfide (MoS_2_/TiS_2_) nanosheet‐based inks achieved an areal capacitance of 448.16 mF cm^−2^, excellent cycling stability of over 100 000 cycles, and energy and power densities of 3.89 and 250 µW cm^−2^, respectively, highlighting their potential for high‐performance microsupercapacitors [86]. Beyond conductivity, MoS_2_ also serves as a photothermal crosslinking center in modular polymer‐nanosheet hydrogel systems, enabling near‐infrared light‐triggered gelation without UV exposure. This system supports conceptual 3D printing and is applicable to various metal disulfides, offering promise for wearable sensors and noninvasive in vivo bioprinting [87]. Furthermore, photothermal properties of MoS_2_ have been leveraged in multifunctional bone‐repair hydrogels. A composite hydrogel incorporating gadolinium complex and MoS_2_ into an *N*‐acryloyl glycinamide/gelatin methacrylate network exhibited enhanced mechanical properties, controllable degradation, and efficient photothermal ablation of tumor cells and bacteria. The gadolinium ions enabled magnetic resonance imaging monitoring and promoted osteogenesis, demonstrating a combined therapeutic, and regenerative strategy for bone tissue engineering [88].

### 3.3 MXene

MXene represents an emerging class of 2D transition metal carbides, nitrides, and carbonitrides. Their nomenclature have general formula M*_n_*_+1_X*_n_* T*_x_*, in which T stands for the surface functional groups, including OH, O, F, Cl, etc., which may differ depending on preparation methods [89]. MXene exhibit exceptional mechanical robustness and electronic conductivity essential for functional hydrogel fillers: monolayer Ti_3_C_2_T*_x_* MXene demonstrates a Young's modulus of ∼0.33 × 10^12^ Pa [90], while its electrical conductivity (6000–8000 S/cm) rivals multilayered graphene and exceeds that of carbon nanotubes and reduced graphene oxide [91]. This synergistic combination of high stiffness and metallic conduction renders MXenes particularly effective for reinforcing mechanically durable, highly conductive networks within 3D printed hydrogel‐based sensors.

A representative study demonstrated the use of direct ink writing for fabricating MXene‐bonded hydrogels as a cost‐effective route to multifunctional sensing, achieving simultaneous strain and temperature detection with a gauge factor of 5.7 (0%–191% strain) and a thermal sensitivity of −5.27% per °C (0–80°C). The resulting 3D printed sensor allowed precision thermal monitoring of shape‐memory solar array hinges in space applications [92]. Further functional versatility was illustrated by a Kirigami‐structured organohydrogel incorporating MXene and sodium alginate nanofibrils, which exhibited exceptional stretchability exceeding 5000%, environmental stability over 30 days, and an ultrahigh gauge factor of 29.1. This system enabled subaquatic human–machine interaction via smart gloves and achieved a machine learning‐assisted Morse code recognition accuracy over 99% [93].

### 3.4 Non‐2D Fillers

In contrast to 2D fillers, non‐2D fillers impart conductivity through alternative mechanisms, each presenting distinct advantages, and limitations. Ionic species, such as ionic liquids (ILs), leverage the hydrogel's hydrated network to enable ion transport, offering high intrinsic conductivity, optical transparency, and biocompatibility, making them ideal for transparent bioelectronics and electrochemical sensors [94, 95]. Conductive polymers, most notably poly(3,4‐ethylenedioxythiophene):poly(styrene sulfonate) (PEDOT:PSS), provide a fully organic and biocompatible alternative with tunable electronic properties, though their integration is often hampered by poor aqueous dispersibility and inherent brittleness; innovative strategies, such as molecular engineering with sulfonated alginate or the development of bicontinuous phases with polyurethane, have been employed to overcome these hurdles [96, 97]. Metallic nanomaterials (e.g., Ag flakes, nanoparticles) offer unparalleled electrical conductivity but face challenges with aggregation, interfacial impedance, and potential compromise of mechanical compliance; they are typically incorporated via preblending, in situ synthesis, or surface metallization to form conductive pathways [63, 98, 99]. The selection among these non‐2D fillers is thus dictated by a trade‐off between achieving high conductivity, maintaining mechanical integrity, and ensuring process compatibility.

**FIGURE 4:**
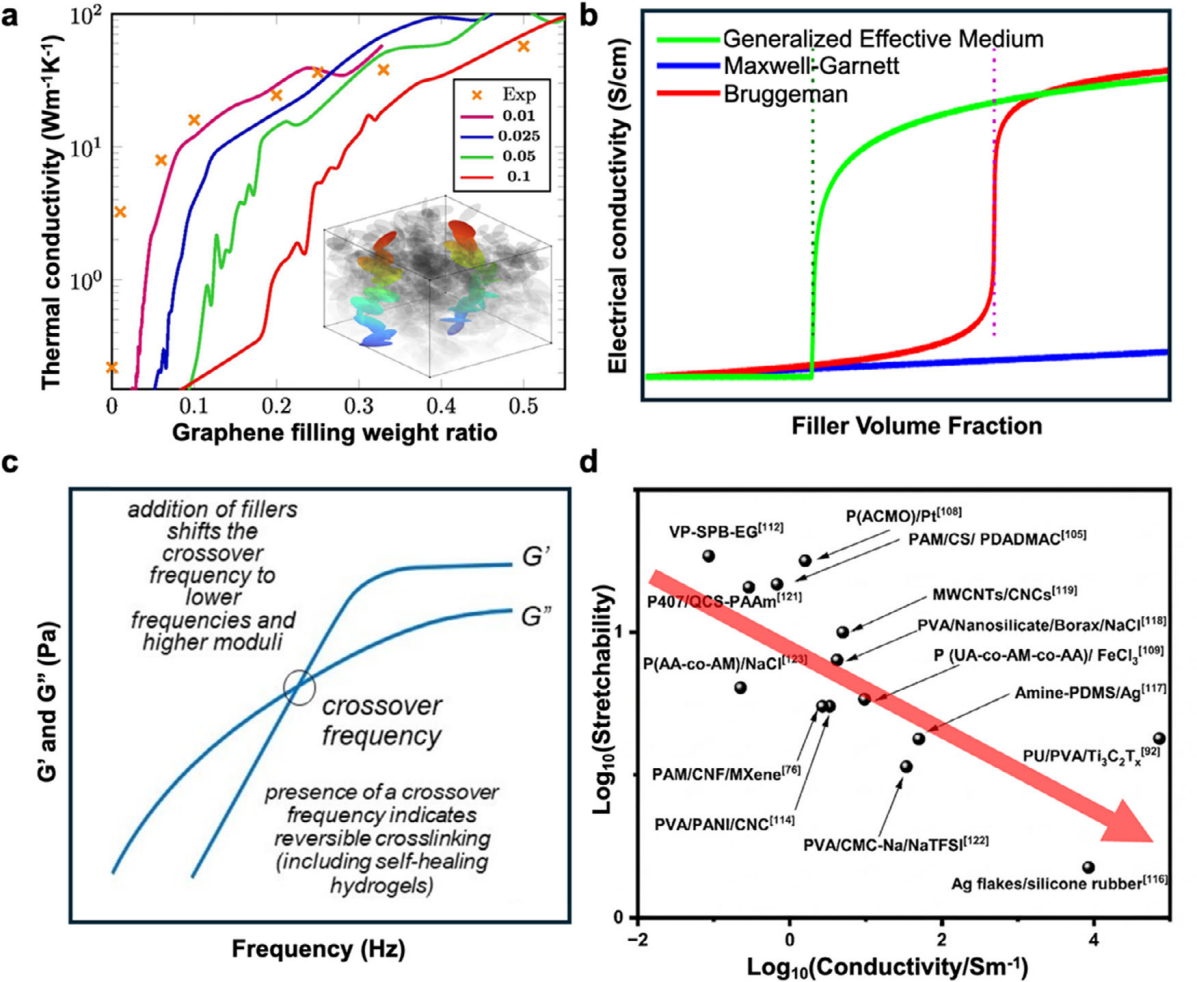
Multiscale structure–property relationships in composite hydrogels with conductive fillers. (a) Thermal conductivity of graphene‐polymer composite vs. the graphene filling weight ratio. Cross: experimental values, solid curves: ellipsoidal simulations with multiple aspect ratios. The inset shows for a randomly generated set of ellipsoids the only unique and most optimum percolation paths. Reproduced with permission [104]. Copyright 2021, Elsevier. (b) Theoretical models predicting the effective conductivity (σ) of the conductive hydrogels. The Maxwell–Garnett model, assuming isolated fillers, predicts a gradual increase without a percolation threshold. The Bruggeman model predicts a sharp transition at a fixed threshold (φ_c_ ≈ 1/3 for spheres). The generalized effective medium (GEM) theory incorporates an adjustable percolation threshold (φ_c_) and critical exponent (*t*). For 2D filler‐based conductive hydrogels (e.g., with MXene or graphene), the GEM model is uniquely capable of accurately describing the low experimental φ_c_ (0.1–2 vol%) and the subsequent nonlinear rise in conductivity, whereas the MG and BG models fail to capture this key behavior. (c) Frequency sweep rheology of composite hydrogels with conductive fillers. The addition of fillers significantly increases both the storage (*G*′) and loss (*G*″) moduli across the entire frequency range. Notably, the crossover frequency (where *G*' = *G*”), indicating the transition from liquid‐like to solid‐like behavior, shifts to a lower value, suggesting the formation of a more robust and elastic network with longer relaxation times. Reproduced under the terms of the CC‐BY license [100]. Copyright 2021, Licensee MDPI, Basel, Switzerland. (d) Conductivity and stretchability of different hydrogel‐based flexible sensors. Mechanically, filler concentrations well above φ_c_ introduce a rigid percolating network that increases the composite's Young's modulus and reduces its fracture strain, thereby degrading the intrinsic softness and high stretchability that are hallmark advantages of hydrogel matrices.

The performance and printability of 3D‐printed hydrogel sensors are fundamentally governed by the percolation behavior of the conductive fillers and their resultant volume fraction. To quantitatively predict and analyze this behavior, several effective medium theories are commonly employed, each with distinct physical assumptions and applicability. The Maxwell–Garnett (MG) model, which treats conductive fillers as isolated inclusions within an insulating matrix, predicts a gradual increase in conductivity with filler fraction but fails to capture the sharp percolation transition. For systems with 2D fillers that readily form interconnected networks at low loadings, the MG model's assumption of noninteracting particles is fundamentally invalid, leading to a severe underestimation of conductivity at practically relevant filler fractions. The Bruggeman (BG) model, by symmetrically treating both phases, can predict a fixed percolation threshold (typically near 1/3 for spherical particles in 3D), yet its threshold is geometry‐dependent and often unrealistically high for anisotropic fillers. When applied to 2D fillers, even adjustments for shape factors yield a predicted threshold (φ_*c*_ ≈ 0.5–0.67) that is still an order of magnitude higher than the experimentally observed range of 0.001–0.02 volume fraction, making it unsuitable for guiding the design of low‐loading, high‐performance composites. In contrast, the generalized effective medium (GEM) theory, also known as the McLachlan equation, incorporates two key parameters: the percolation threshold (φ_c_) and the critical exponent (*t*). Its general form reduces to a power‐law, σ∝(φ − φ_c_)^*t*^ for φ > φ_c_ under the condition σ_filler_ ≫ σ_matrix_, providing unparalleled flexibility to accurately describe the nonlinear conductivity jump near φ_c_ and the subsequent growth for diverse composite systems. This adaptability is why the GEM theory is uniquely powerful for modeling composites with 2D fillers. Its core parameter φ_c_ can be fitted to the exceptionally low experimental thresholds (e.g., 0.1–2 vol%) characteristic of 2D materials, while the exponent *t* captures the dimensionality of the conductive network, thereby offering a quantitatively accurate framework for predicting and optimizing the conductivity of printed hydrogel sensors as shown in Figure 4b.

The viscoelastic behavior of the hydrogel inks, crucial for assessing their printability and structural stability, was characterized through oscillatory frequency sweeps. As shown in Figure 4c, the incorporation of conductive fillers profoundly alters the rheological response. Both the storage modulus (*G*′), representing the elastic component, and the loss modulus (*G*″), representing the viscous component, exhibit a substantial increase across the measured frequency range. This universal enhancement indicates an overall reinforcement of the composite network. The most critical observation is the downward shift of the crossover frequency (ω_c_, where *G*′ = *G*″) with increasing filler content [100]. In a pure hydrogel, the crossover at a higher frequency signifies a relatively fast relaxation process where viscous flow dominates over longer timescales. The shift to a lower ω_c_ in the composites implies that the embedded fillers—through physical interactions, confinement, or the formation of a percolating network—effectively restrict the long‐range motion of polymer chains. This dramatically slows down the relaxation dynamics, extending the timescale required for the material to flow. Consequently, the composite exhibits a more pronounced solid‐like, elastic character at lower frequencies, which is a key indicator of enhanced structural integrity and shape retention, directly benefiting processes like direct‐ink‐writing (DIW) 3D printing. Moreover, the percolation threshold (φ_c_) is the critical filler volume fraction at which a continuous conductive network forms, leading to a dramatic, nonlinear increase in electrical conductivity. Achieving conductivity necessitates a filler loading at or above φ_c_. However, exceeding the optimal loading introduces a series of trade‐offs that adversely affect both processability and mechanical properties. For DIW, high filler content sharply increases the ink's viscosity and yields stress, which can impair its shear‐thinning behavior, lead to nozzle clogging, and ultimately restrict the printable resolution and geometric complexity. In DLP and SLA, the incorporation of nanofillers, particularly those with high aspect ratios, scatters and absorbs UV light, significantly reducing the curing depth and polymerization rate. This necessitates careful cooptimization of photoinitiator concentration, exposure time, and filler loading to avoid incomplete curing or interlayer delamination. Mechanically, filler concentrations well above *φ*_c_ introduce a rigid percolating network that increases the composite's Young's modulus and reduces its fracture strain, thereby degrading the intrinsic softness and high stretchability that are hallmark advantages of hydrogel matrices as shown in Figure 4d.

Owing to their ultrahigh aspect ratio and large specific surface area, conductive networks formed by 2D fillers can achieve electrical percolation at very low volume fractions. For instance, a direct experimental comparison of carbon‐based fillers demonstrated that graphene achieved superior conductivity at a given loading compared to carbon black (0D) and carbon nanotubes (1D), attributable to its more efficient formation of conductive pathways [101]. Theoretical work further confirms that fillers with higher specific surface area provide greater interfacial contact and more effective electron tunneling sites per unit volume, making them intrinsically more efficient for constructing conductive networks in composites [102]. Consequently, typical percolation thresholds for MXenes and graphene derivatives range from 0.1 to 2 vol%, significantly lower than the 5–20 vol% required for 0D nanoparticles or 1D nanowires. A minimal loading of 1–3 wt% of a 2D filler can thus achieve high conductivity (>1000 S/m for MXenes) while largely preserving the hydrogel's mechanical compliance and stretchability (>500%). This low φ_c_ also widens the printability window by mitigating the negative rheological and optical effects associated with high filler content.

The relationship between filler fraction (φ) and composite conductivity (σ) above the percolation threshold is described by the power‐law percolation model: σ∝(φ − φ_c_)^*t*^, where *t* is the critical exponent. For a 3D random network of spherical particles, *t* ≈ 2.0, but this value decreases for fillers with high aspect ratios [103]. This geometric effect explains the steeper rise in conductivity near φ_c_ for 2D fillers. This model underscores that conductivity increases rapidly just above φ_c_, but further gains require disproportionately larger filler additions, which intensify the aforementioned trade‐offs. Guidance for selecting filler loadings can be derived from these principles. For applications prioritizing maximized softness and stretchability (e.g., epidermal sensors), using 2D fillers with loadings slightly above φ_c_ is optimal. For applications requiring ultrahigh conductivity (e.g., interconnects), higher loadings can be used, accepting a moderate increase in modulus, potentially enhanced by alignment strategies in DIW or EHD. Ultimately, the loading must satisfy the rheological demands of the chosen printing technique. In summary, the strategic selection of low‐percolation‐threshold 2D fillers and the precise tuning of their volume fraction, guided by percolation theory, is paramount for navigating the trade‐off triangle of conductivity, mechanical compliance, and printability.

## 4 Microstructures Enhanced Hydrogel‐Based Sensors

Designed with geometries including pyramidal [124, 125], porous [126, 127], hemispherical [128, 129], and cylindrical [130, 131] microstructures have been a crucial strategy for enhancing the performance of flexible sensors. The methodology has been extended to hydrogel‐based sensors fabricated via 3D printing technologies, where controlled microstructural design offers promising avenues for performance optimization. However, inherent limitations in 3D printing resolution and hydrogel material properties impose constraints on replicating certain natural microstructures, such as leaf‐inspired patterns [132] or ultrafine fibrous networks [133]. Through comprehensive analysis of current research, this section focuses specifically on two predominant and manufacturable microstructural categories in 3D‐printed hydrogel sensors: microporous structures and micropatterns, which have demonstrated effectiveness in balancing fabrication feasibility with enhanced sensor performance.

### 4.1 Microporous Structure

**FIGURE 5:**
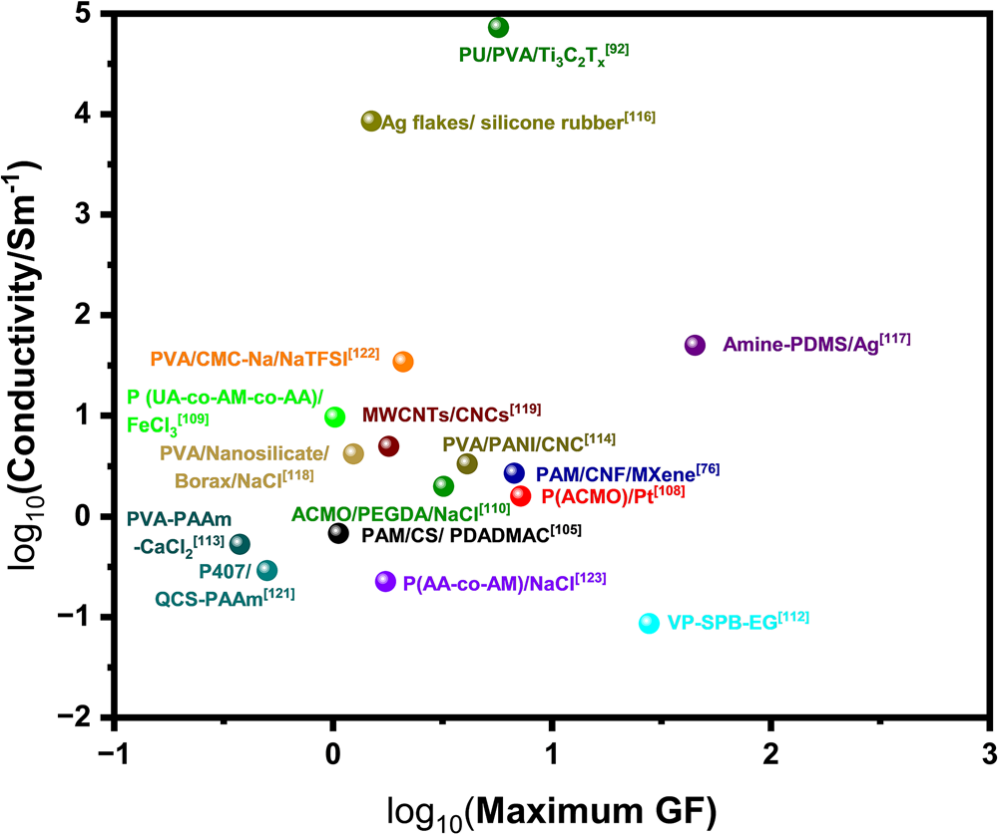
Hydrogel‐based flexible sensors‐fabricated by 3D printing. Maximum GF and conductivity of different kinds of hydrogel‐based flexible sensors.

**FIGURE 6:**
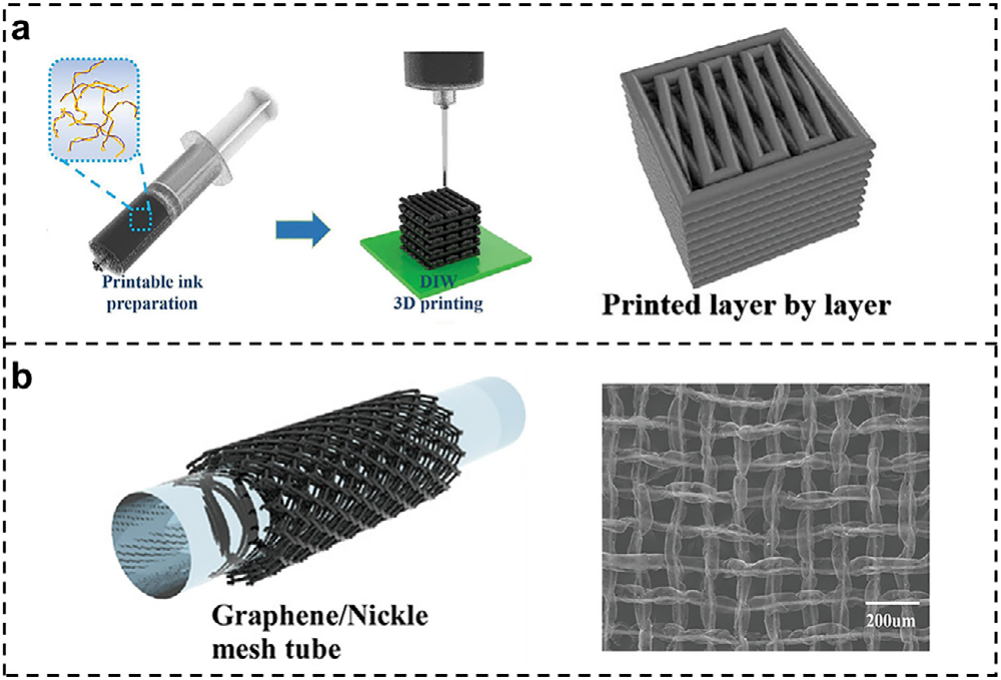
Hydrogel‐based flexible sensors with microporous. (a) WPU/SWCNT/CNF sponge by DIW printing and its 3D grid structure. Reproduced under the terms of the CC‐BY license [137]. Copyright 2024, Wiley‐VCH GmbH. (b) Graphene/nickel mesh by CVD growth and its SEM image. Reproduced with permission [141]. Copyright 2022, Wiley‐VCH GmbH.

The layer‐by‐layer deposition characteristic of 3D printing inherently facilitates the fabrication of microporous structures, making them the predominant structural configuration in hydrogel‐based sensors (Figure 5). The microporous structures in 3D‐printed hydrogel sensors fundamentally enhance performance by creating stress concentration effects that amplify deformation‐induced signal changes while simultaneously enabling dynamic contact area modulation for broad‐range linear responses. Additionally, these interconnected porous networks contribute to optimizing ion transport, achieving tunable sensitivity through precise pore size control. The spatial microporous structure significantly enhances the performance of hydrogel‐based tactile flexible sensors. These interconnected cellular configurations exhibit superior compressibility compared to conventional solid structures, which can enhance the structural integrity during repeated compression cycles. Previous work successfully fabricated a highly compressible 3D‐printed porous tactile sensor, where complex stretchable 2‐hydroxyethyl acrylate‐isobornyl acrylate and annealed highly conductive PEDOT:PSS were adopted [134]. The sensor exhibited highly reproducible resistance responses during cyclic compression tests across a wide strain range from 20% to 80%. Similarly, another group developed a novel flexible sensor with microporous structure based on a composite of modified MXene nanosheets and photocurable polyurethane acrylate resin matrix [135]. The sensor maintains structural integrity without damage after 200 compression cycles at 60% strain. The microporous based flexible sensor reported by some groups demonstrated a sensitivity of 280 Pa^−1^ at 60% compressive strain with rapid response/recovery times of 298/348 ms [136]. Different from abovementioned sensors, one novel work developed an anisotropic tactile sensor illustrated in Figure 6a and sensitivity reached 1.22 (kPa wt%)^−1^ across a broad pressure range of 2.8–8.1 × 10^3^ Pa by introducing microscopic pore morphology [137].

The incorporation of microporous structures into strain flexible sensors represents a significant advancement in enhancing their tensile characteristics. This structural modification enables the sensor to accommodate greater elongation while maintaining electrical conductivity through the formation of continuous conductive pathways along the pore walls. The porous network effectively reduces stress concentration points that typically lead to premature fracture in conventional solid structures. A 2 × 2 cm^2^ grid‐patterned conductive layer printed from a silk‐fibroin‐based conductive hydrogel exhibited stable performance over >1000 tensile cycles [138]. In another study, researchers fabricated lattice, circuit, and tubular structures with well‐defined microporous structures using polyaniline‐based inks through 3D printing, achieving an exceptional strain sensing range of 0%–764.4% with a gauge factor of 1.4 [139]. Similarly, a double‐network hydrogel (sodium alginate/polyvinyl alcohol in a glycerol–water medium) realized complete shape recovery after 300% tensile strain held for 1 min [140]. In a separate application, a graphene‐based nerve guidance conduit incorporated a natural double‐network hydrogel and a neurotrophic concentration gradient (Figure 6b) [141].

### 4.2 Micropatterns

The introduction of 3D micropatterns on flexible sensor surfaces can enable controlled elastic deformation of the contact surface. This deformation mechanism promotes reversible energy storage and release during operation, which effectively reduce viscoelastic losses in the material. Consequently, the sensor achieves optimized dynamic response characteristics, and the structural integrity remains preserved even under repeated mechanical loading cycles. Traditionally, the fabrication of microstructure usually depends on the molds manufactured by photolithography. However, photolithography incurs high costs, and the molding method exhibits resistance to arbitrary microstructures, which may impede the rapid prototyping of hydrogel‐based sensors. With the improvement of printing resolution and features precision, 3D printing technologies have become an effective method for manufacturing arbitrary microstructures.

#### 4.2.1 Biomimetic Micropatterns

**FIGURE 7:**
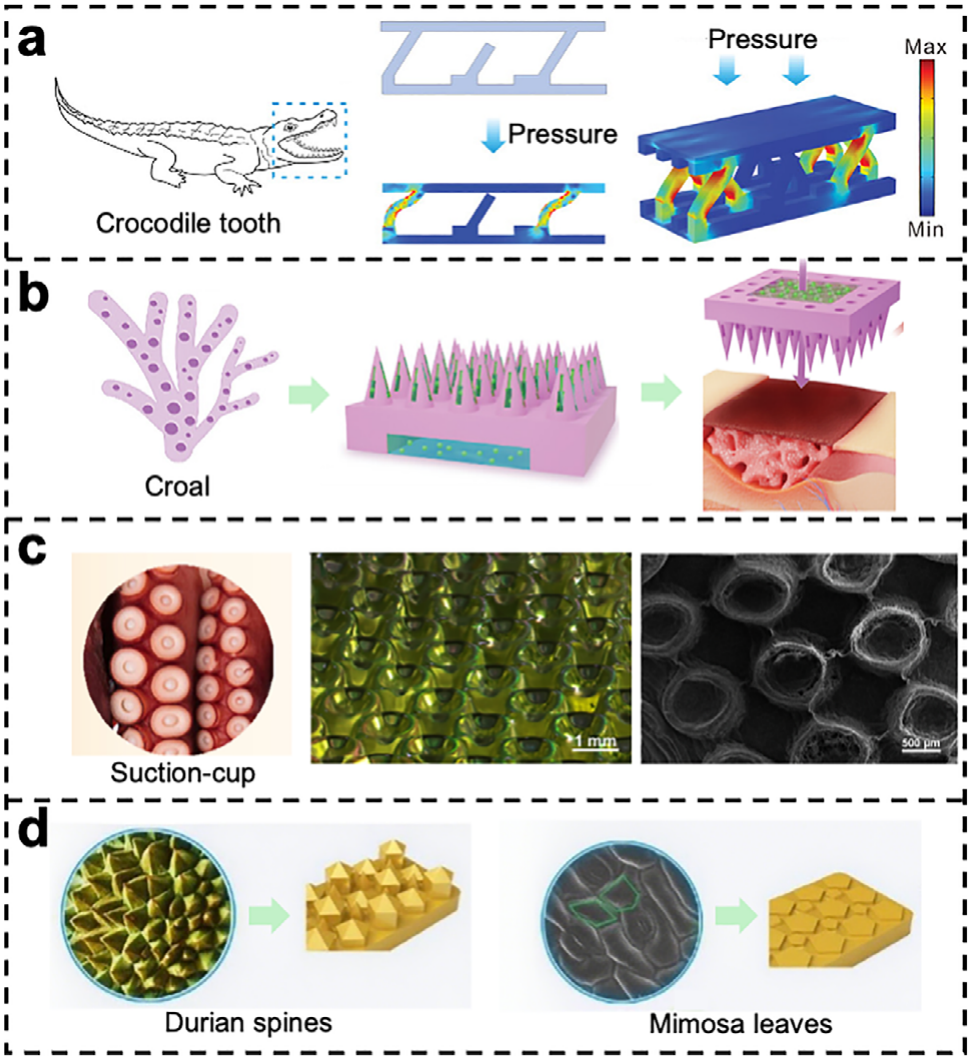
Hydrogel‐based flexible sensors with biomimetic micropatterns. (a) Pressure sensors inspired by the structure of crocodiles. Reproduced with permission [143]. Copyright 2023, Wiley‐VCH GmbH. (b) Design inspiration for porous and hollow microneedles derived from the structure and biological behavior of corals. Reproduced with permission [144]. Copyright 2024, Wiley‐VCH GmbH. (c) SEM image, adhesion and detachment mechanism and relative resistance versus strain curves of PA and PAMS hydrogels inspired by octopus sucker structures and snail mucus. Reproduced with permission [145]. Copyright 2024, Acta Materialia Inc. (d) Schematic illustration of the bionic coupling structure design, along with finite element method simulation results. Reproduced with permission [146]. Copyright 2024, Elsevier B.V.

Microstructures are widely presented in biological systems, such as the micro/nano structures on lotus leaves, the microscopic setae on gecko feet and the bristles on spider legs. These naturally evolved microstructures play a crucial role in improving the environmental sensing capacity of organisms. Hence, by mimicking these microstructures and integrating them into flexible sensors, the sensitivity, durability, and environmental adaptability can be improved significantly. To overcome the limitations of traditional photolithography in replicating biological microstructures, 3D printing technologies have been employed for direct fabrication of biomimetic architectures. Fingerprint‐mimicking hydrogel‐based tactile sensors have been successfully developed through 3D printing of epidermal microstructures, replicating the sophisticated ridge‐valley architecture of human fingerprints and achieving remarkable sensing performance with a sensitivity of 60 Pa^−^^1^ across a broad detection range from 26 Pa to 7 × 10^4^ Pa [59]. In a similar biomimetic approach, intestinal villi‐like microstructures were fabricated on screen‐printed electrodes using 3D printing technology, demonstrating a wide linear detection range of 0.1–0.8 ng/mL and an impressively low detection limit of 0.036 ng/mL [142]. Drawing inspiration from the misaligned tooth arrangement of crocodiles, a capacitive pressure sensor with graded filling structure was designed, exhibiting high sensitivity up to 970 Pa^−^^1^ and a wide pressure detection range from 7 to 3.8 × 10^5^ Pa (Figure 7a) [143]. Furthermore, coral‐inspired microneedle patches have been fabricated through 3D printing technology, where each microneedle featured uniform porous shells and hollow cavities that were subsequently loaded with heparin‐based functional hydrogels, enabling autonomous regulation of antimicrobial release speed based on feedback from integrated sensors [144].

Furthermore, the integration of hierarchical biomimetic microstructures has emerged as a significant strategy for enhancing the performance of flexible sensors. For instance, drawing inspiration from octopus suckers and snail mucus adhesion mechanisms, a hydrogel‐based sensor with multicoupled biomimetic structures was developed that achieves 460% strain deformation while maintaining a gauge factor of 4.73 (Figure 7c) [145]. In another approach, hierarchical micropatterns mimicking mimosa leaves, durian spikes, and octopus suckers were fabricated on hydrogel‐based flexible sensor surfaces, resulting in a 91.22% reduction in deformation and 57.5% enhancement in adhesion strength compared to unstructured counterparts (Figure 7d) [146].

#### 4.2.2 Geometric Patterns

**FIGURE 8:**
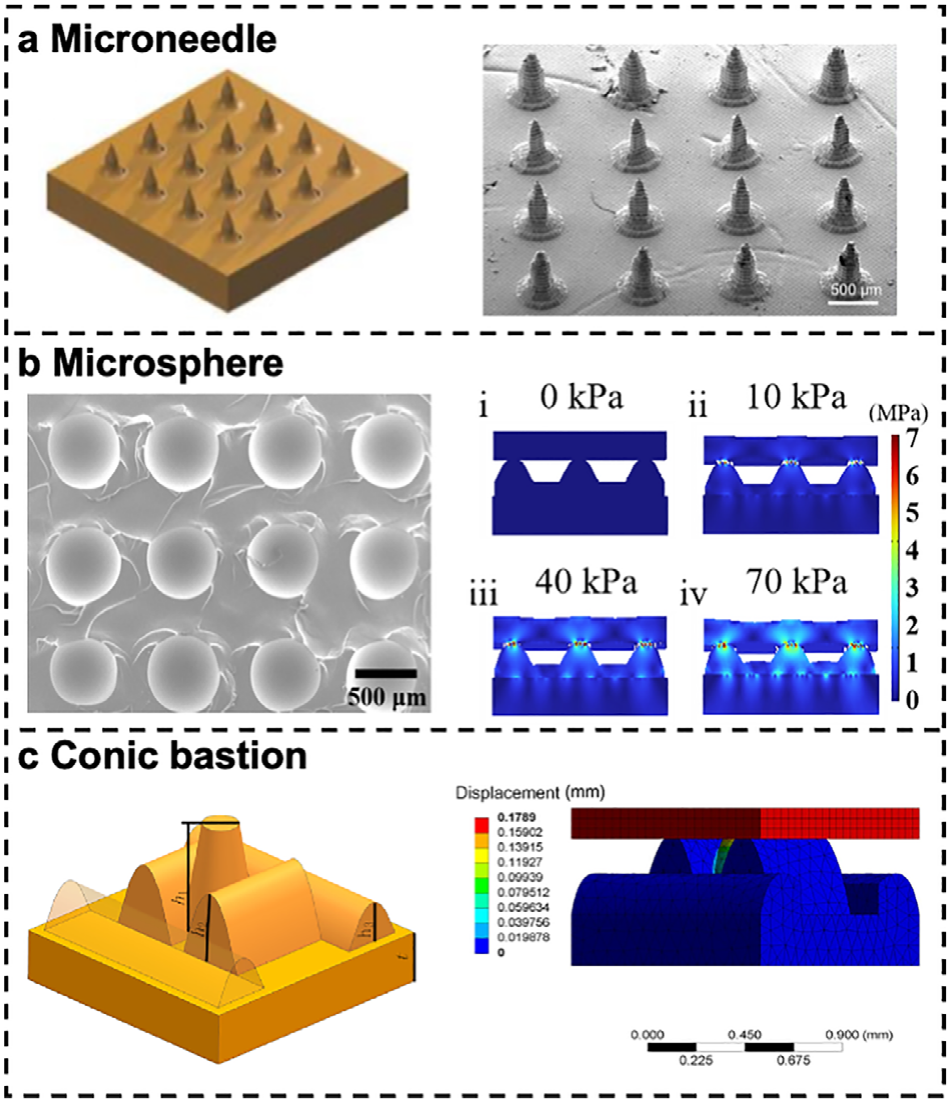
Hydrogel‐based flexible sensors with geometric micropatterns. Schematic illustration, morphology, simulation, and characterization of 3D‐printed hydrogels with (a) Microneedle arrays fabricated by DLP from crosslinked poly(ethylene glycol) diacrylate (PEGDA). Reproduced under the terms of the CC‐BY license [148]. Copyright 2023, Licensee MDPI, Basel, Switzerland. (b) Spherical microstructures fabricated from acrylamide and PVA. Reproduced under the terms of the CC‐BY license [150]. Copyright 2025, Springer Nature. (c) Conic bastion structure fabricated by DLP from acrylamide and PEGDA. Reproduced with permission [151]. Copyright 2024, Wiley Periodicals LLC.

Microcones show significant capacity in improving the performance of 3D printed hydrogel‐based sensors due to their enhanced interfacial contact and stress concentration effects. The sharp conical tips generate localized stress concentration, thereby significantly enhancing sensitivity to minute deformation or pressure. The graded multilevel structure of the microcones effectively dissipates stress, mitigating material fatigue, and is particularly suitable for high‐precision strain sensing applications [144]. This advantage was clearly demonstrated in a study where digital light processing was employed to construct eutectic gallium‐indium microcone arrays, achieving an 8.78‐fold enhancement in detection sensitivity compared to traditional sheet structures [147]. Beyond sensing applications, 3D printed hydrogel microcone arrays have also been developed for rapid interstitial fluid biomarker extraction and colorimetric detection, enabling complete biomarker identification within minutes (Figure 8a) [148]. Further expanding their utility, a solvent‐free ionic elastomer‐based pressure sensor featuring 3D printed microcone arrays exhibited markedly superior signal responsiveness compared to nonpatterned sensors across varying applied pressures [149].

Spherical microstructures in flexible sensors can achieve wide‐range pressure detection through controlled radial compression, maintaining structural integrity and breathability for epidermal applications. Furthermore, the spherical protrusions form a uniform contact interface, enabling conformal contact with complex curvilinear surfaces while minimizing shear stress interference. A flexible sensor integrating sandpaper‐molded microstructures with resonantly printed interdigitated electrodes demonstrated exceptional tunable sensitivity from 5.8 to 24 Pa^−^^1^ across a broad detection range of 1–100 × 10^3^ Pa [152]. Another microsphere‐enhanced hydrogel‐based multimodal sensor achieved outstanding pressure sensitivity of 30.6 × 10^3^ Pa^−^^1^ alongside temperature sensitivity of 0.5°C^−1^ (Figure 8b) [150]. Furthermore, an adaptable biosensing platform featuring versatile circular electrodes integrated with flexible 3D printed wristband structures was validated through electrocardiography (ECG) and electromyography (EMG) measurements, confirming its efficacy in real‐world applications [153].

Additionally, the incorporation of irregular microstructures has emerged as another effective strategy for enhancing the performance metrics of hydrogel‐based flexible sensors. For instance, a 3D printed hydrogel sensor with gradient hierarchical structures achieved a sensitivity of 43 Pa^−^^1^, representing a ninefold enhancement compared to conventional planar designs [154]. In another study, a multiscale structural design in 3D printed hydrogel sensors yielded a fiftyfold sensitivity improvement over nonstructured counterparts [155]. Similarly, the introduction of conical bastion microstructures into hydrogel sensors resulted in exceptional dynamic performance with 30 ms response and 40 ms recovery times (Figure 8c) [151].

Despite significant progress in architected hydrogels, current studies overwhelmingly rely on qualitative descriptions without identifying the governing geometric parameters or providing predictive models. For example, the linear sensing range of microporous pressure sensors is dominated not by porosity alone but by pore aspect ratio, ligament slenderness, and local buckling modes—parameters rarely reported in the literature. Similarly, bio‐inspired surface micropatterns exhibit strong anisotropic responses, yet most works do not quantify hierarchical effects. The absence of design rules is becoming the field's primary bottleneck. Integrating machine learning guided microstructure optimization design represents a crucial opportunity for the next stage of development.

## 5 Application of 3D Printed Flexible Sensors

Hydrogel‐based flexible sensors have attracted many attentions due to their tissue‐like mechanical properties, excellent biocompatibility and tunable functionalities [156, 157, 158]. The advancements in material engineering and microstructure design have substantially improved their sensitivity, detection range, and durability [159, 160]. The hydrogel‐based sensors have demonstrated remarkable applicability in continuous monitoring of physiological signals as well as for tracking complex body movements. In addition, the capacity of precise tactile feedback and environmental interaction shows the potential application in the fields of robotic manipulation and food safety detection.

### 5.1 Human Motion Detection

**FIGURE 9:**
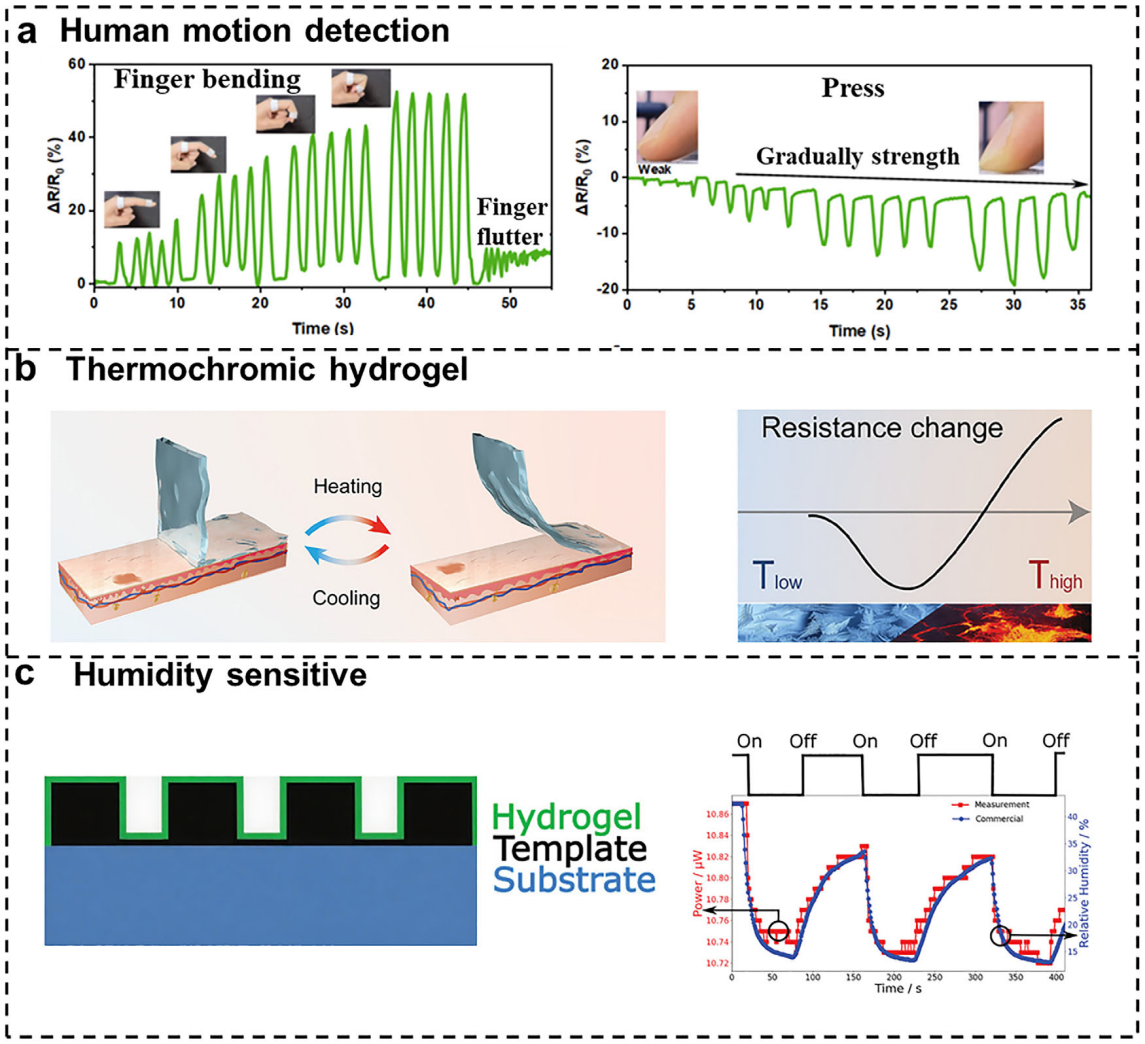
Applications of hydrogel‐based flexible sensors for human motion and environmental detection. (a) The relative resistance change of different human motions. Reproduced with permission. [167] Copyright 2025, Elsevier Ltd. (b) Mechanism and response of thermos responsive hydrogels. Reproduced with permission. [185] Copyright 2025, Wiley‐VCH GmbH. (c) Ultrathin hydrogels deposited by CVD and their response under humidity conditions. Reproduced under the terms of the CC‐BY license. [186] Copyright 2023, Wiley‐VCH GmbH.

Considering the high stretchability and mechanical compliance matching human tissue, hydrogel‐based flexible sensors exhibit exceptional applications in human motion detection. The monitoring of finger, wrist, elbow, neck, ankle movements, and even facial muscle contractions constitutes critical biomechanical signals routinely assessed in medical health applications [161, 162, 163]. Different from traditional sensors, the abovementioned properties permit robust interfacial contact between flexible sensors and human dynamic body movements ensuring the measurement precision. Some researchers applied the DIW 3D printed carbon nanotube incorporated composite hydrogel‐based sensor to monitor both joint movements and facial expressions [120]. Similarly, a capacitive hydrogel‐based flexible sensor made by DIW method was reported, which can be used to monitor joint bending [164]. In another study, as shown in Figure 9a, the correlation between human hand gestures and the corresponding output electrical signal have been systematically investigated by wearing hydrogel‐based sensors [165, 166, 167]. The established fundamental mapping relationships not only hold significant potential for facilitating communication among the hearing‐impaired population but also enable accurate gesture recognition in emergency situation. More significantly, Wu et al. developed an intelligent gesture recognition system by integrating artificial neural networks with machine learning algorithms.

In addition, remote precise control of bionic robotic hands demonstrates significant potential for performing complex operations in extreme environments or assisting individuals with hand injuries in daily activities. Recent demonstrations employ DLP 3D‐printed hydrogel sensors to capture multichannel electrical signals and, via a tailored encoding/decoding scheme, map them to finger‐specific commands, enabling accurate, repeatable remote manipulation of bionic hand movements [168, 169].

### 5.2 Human Physiological Signal

Unlike the obvious deformations caused by human motion, physiological signals typically induce only weak strain in flexible sensors, which provides high requirement for detection sensitivity of hydrogel‐based sensors. To achieve high‐fidelity acquisition of subtle bio‐signals, such as pulse beat or muscle tremors, researchers have reported some approaches to improve signal fidelity, including microstructures to amplify strain‐responsive signals, development of advanced functional filling materials and integration of machine learning algorithms [170].

ECG (electrocardiography) is a vital diagnostic modality that records cardiac electrical activity and enables comprehensive assessment of rhythm and conduction, aiding the identification of arrhythmias and myocardial infarction. A fully flexible ECG system featuring easily detachable hydrogel electrodes was reported to overcome limitations of conventional Ag/AgCl electrodes [171]. Coupled with a convolutional neural network, it classified supine, sitting, and standing postures with 97.9% accuracy. In another study, integrating conductive hydrogel with nonwoven fabric yielded an ECG electrode that maintained reliable signal acquisition under both static and dynamic conditions, effectively mitigating motion artifacts while preserving signal fidelity [172]. To enhance sensitivity, pyramid microstructures were introduced at the electrode‐skin interface, enabling stable long‐term ECG monitoring [173]. The developed sensor can be used to monitor ECG for a long time.

EMG (electromyography) provides a quantitative assessment of neuromuscular function by recording electrical muscle activity, which offers objective metrics to monitor pathological changes and therapeutic progress in neuromuscular disorders. One research group printed a novel disc‐shaped hydrogel electrode for EMG monitoring, which demonstrated superior performance with a signal‐to‐noise ratio of 32.1 ± 2.3 dB when recording tibialis muscle activity, representing a 50% improvement over conventional graphene/agarose electrodes [174]. Another demonstrated that the DIW 3D‐printed hydrogel sensor could precisely detect EMG of rabbit leg under varying electrical stimulation intensities [175]. This technology can effectively prevent both overtreatment‐induced tissue damage and insufficient stimulation.

3D‐printed hydrogel sensors also show strong potential for integrated cardiorespiratory monitoring in clinical settings. A high‐sensitivity biosensor was formulated by DIW printing egg‐white cross‐linked protein hydrogels doped with conductive nanomaterials, enabling accurate acquisition of both pulse signals and respiratory rate [176]. A customizable metamaterial, gelatin‐based conductive membrane simultaneously tracked cardiac activity and respiratory patterns by transducing subtle cardiac‐induced deformations [177]. For breath‐based health assessment, a portable 3D‐printed acetone‐sensing platform provided dual‐mode readout—visual colorimetry and quantitative luminescence—for detecting exhaled acetone [178].

### 5.3 pH Detecting

When the environmental pH changes, the functional groups (such as carboxyl and amino groups) within the hydrogel undergo protonation/deprotonation reactions with H^+^, altering the ionic concentration and charge distribution inside the gel. This physicochemical change modulates the hydrogel's electrical conductivity, dielectric constant, or swelling degree, which is subsequently detected in real time through variations in electrical resistance, capacitance, or optical signals. Hydrogel‐based flexible pH sensors exhibit dual functionality for both biomedical and environmental monitoring applications, ensuring precise measurement of physiological pH levels in biological fluids, such as saliva for health assessment, while simultaneously serving as reliable tools for environmental pH detection to acquire critical ecological information.

In biomedical sensing, a DIW printed rapid‐response colorimetric hydrogel for salivary monitoring employed a sodium alginate‐polyvinylpyrrolidone matrix loaded with pH‐sensitive indicators, achieving ∼1 s detection with excellent reproducibility [179]. The sensor can complete detection in a second and possess excellent reproducibility. For portability, a DIW 3D nanomaterials‐printed wearable, battery‐free, and wireless pH sensor used for detection of disease was proposed. This device demonstrates high reproducibility excellent flexibility and outstanding biocompatibility in a pH range from 3.0 to10.0 [180]. In addition, smartphone‐based visualization of skin pH was enabled by integrating a DLP 3D‐printed re‐entrant auxetic hydrogel with colorimetric indicators, allowing noninvasive tracking of healing progression [181].

For environmental monitoring, a DIW 3D‐printed hydrogel pH sensor with enhanced mechanical robustness overcame the stiffness and toughness limitations of cast hydrogels, reaching a toughness of 2.37 MJ m^−^^3^ and delivering high sensitivity across diverse aqueous media [182]. To address adaptability, portability, and environmental compatibility constraints, hydrogel pH sensing combined with machine‐learning algorithms achieved rapid, stable, and reversible detection across pH 4–10 with >99% accuracy, recall, and F1‐score [183].

### 5.4 Temperature and Humidity Sensors

Hydrogel‐based flexible temperature sensors operate through thermos responsive polymer phase transitions, exhibiting reversible optical/electrical property changes. The integration of temperature sensing capability into hydrogel‐based flexible sensors plays a pivotal role in both human disease diagnostics and robotic environmental interaction. To overcome the rigidity, brittleness, and wearability‐induced artifacts of conventional metal/semiconductor sensors, a DIW 3D‐printed porous hydrogel architecture was engineered to enhance temperature‐sensing performance [184]. For real‐time body‐temperature monitoring, a DIW 3D‐printed thermochromic hydrogel based on an *N*‐isopropylacrylamide/acrylic‐acid copolymer (Figure 9b) exhibited reversible transparency shifts with temperature and was leveraged to build an integrated optical alarm system [185]. Distinctly, in situ temperature‐variable Raman spectroscopy was used to reveal a thermally induced tunneling mechanism governing hydrogel temperature response, yielding a sensitivity of −5.27% °C^−1^ across 0–80°C [92].

Hydrogel‐based flexible humidity sensors function through moisture‐induced swelling/deswelling of hydrophilic polymer networks. Water molecule absorption alters intermolecular hydrogen bonding, causing dimensional changes that modulate electrical capacitance/resistance or optical properties. Humidity measurement has been a critical parameter with extensive applications across multiple domains. In industrial settings, it plays a vital role in fuel cell technology for automotive applications, corrosion monitoring, pulp, and paper production, as well as biogas processing. Equally importance are its biomedical implications for human health monitoring and assessment. A thin‐film hydrogel humidity sensor (Figure 9c) with nanostructured features fabricated was proposed by two‐photon polymerization 3D printing [186]. The results indicate that the 50 nm thick sensor exhibits the fastest response time of less than 8 s. Complementing ambient humidity detection, a multimaterial SLA 3D‐printed anisotropic liquid‐leakage sensor enabled localization and directional flow readout, offering early‐warning capability for moisture ingress in complex systems [187]. Further studies realized real‐time environmental humidity monitoring by tracking humidity‐dependent shifts in capacitance and resistance in hydrogel devices [188, 189].

### 5.5 Glucose and Wounds Detection

**FIGURE 10:**
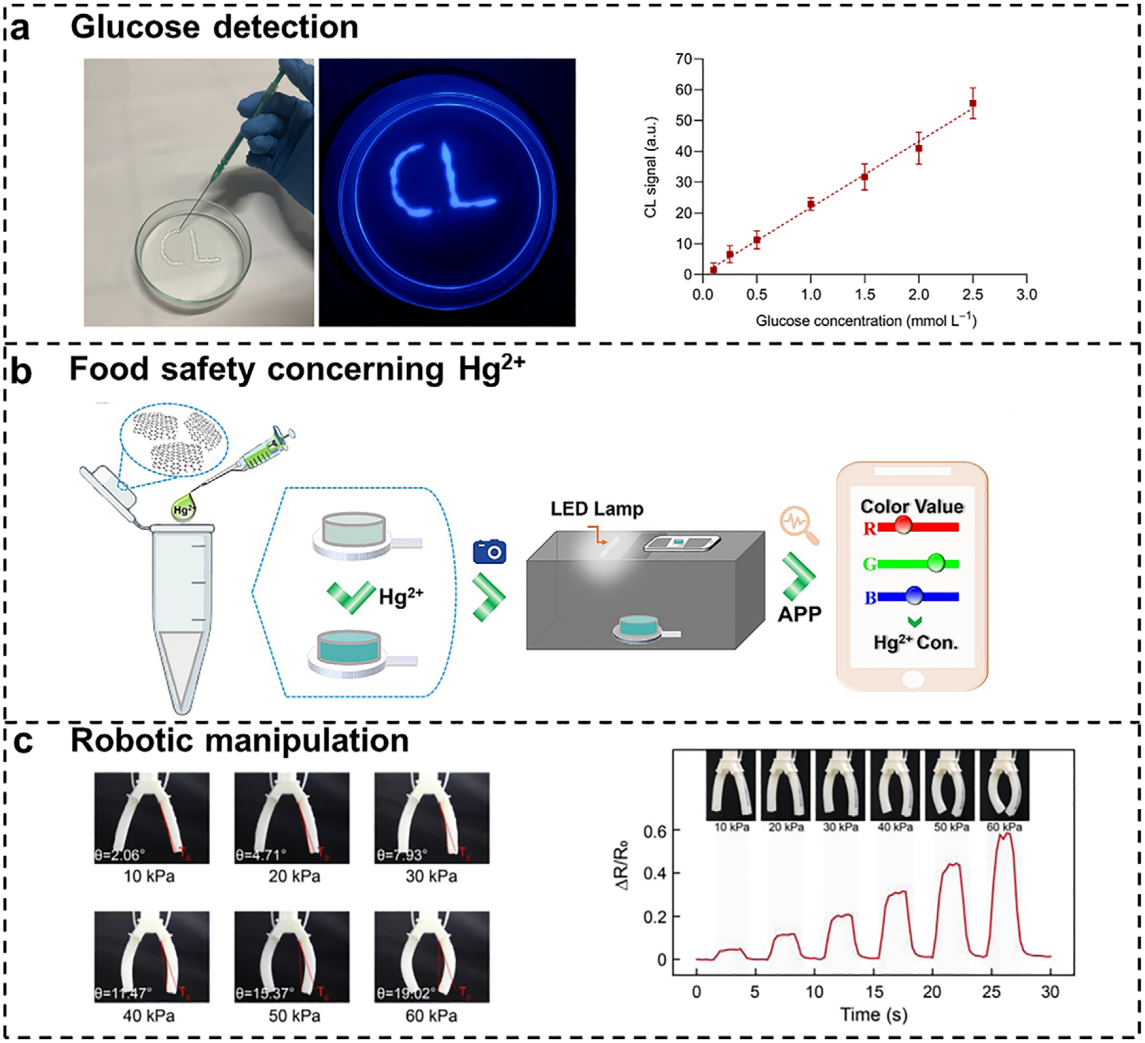
Applications of hydrogel‐based flexible sensors for health detection and robotic manipulation. (a) 3D printing process of the guanosine/hemin chemiluminescent hydrogel using a syringe. Reproduced under the terms of the CC‐BY license [192]. Copyright 2023,  Licensee MDPI, Basel, Switzerland. (b) Procedure of Hg^2+^ concentration detection by the color value. Reproduced with permission [197]. Copyright 2025, Elsevier B.V. (c) Graph showing the relative change in resistance of robotic skin as pneumatic pressure is incrementally increased. Reproduced with permission [201]. Copyright 2022, Wiley‐VCH GmbH.

Rapid and reliable monitoring of blood glucose concentration plays a pivotal role in early warning and prevention strategies for diabetes management. The latest DLP 3D printed hydrogel‐based flexible sensors demonstrate superior performance in real‐time glucose detection, marking a significant advancement in noninvasive monitoring solutions. A label‐free optical design employing a concanavalin A‐based hydrogel achieved a sensitivity of 0.206 nm/mM across 0–25 mM with excellent linearity [190]. In another study, a miniaturized fused deposition modeling 3D‐printed electrochemiluminescent platform using luminol/H_2_O_2_ chemistry and carbon‐black‐doped polylactic‐acid electrodes detected glucose up to 5 mmol L^−^^1^ with a limit of detection (LOD) of 60 µmol/L [191]. Building upon the previous work, the sensor performance was further enhanced by developing a novel self‐assembled guanosine‐derived hydrogel incorporating both luminol and hemin chloride (Figure 10a) [192]. The material exhibited remarkable peroxidase‐mimicking activity toward H_2_O_2_ detection while representing exceptional enzymatic stability and catalytic efficiency even under harsh alkaline and oxidative conditions, achieving improvement of detection limit to 120 µmol/L^−1^.

Wound healing represents a complex physiological process involving intricate tissue regeneration and dynamic interactions between bacterial colonization and immune responses. Throughout this process, dynamic changes in cutaneous acidity, protein composition, and blood components serve as critical indicators of healing progression. The inability to monitor these biochemical parameters in real‐time may lead to subclinical infections developing beneath the wound eschar, potentially compromising the healing outcome. To solve this problem, Tsegay and co‐workers reported a wound monitoring sensor by integrating paper‐based colorimetric device with DLP 3D‐printed auxetic hydrogel wound dressings [193]. This hybrid sensor enables detection of pH and glucose concentration through distinct colorimetric responses across physiologically relevant ranges, which can assist in preventing escalation of the acute wounds into chronic stages in diabetics. In parallel, a high‐precision DLP 3D‐printed functional hydrogel patch was fabricated with heparin loaded into porous microstructures; embedded sensors detected infection status and triggered autonomous drug release, yielding an ∼200% acceleration of infected‐wound healing [145].

### 5.6 Food Safety

Microbial‐induced food spoilage during transportation and storage remains a major contributor to global food waste, while real‐time monitoring of food deterioration lacks effective implementation. While conventional detection techniques, such as UV‐fluorescence probe and visible spectroscopy, have been well‐established, the reliance on complex instrumentation, time‐consuming, and high cost limits their utility. Recently, 3D printed hydrogel‐based sensors offer an essential approach to ensure food safety and mitigate food waste. For instance, an alginate‐gelatin‐nanocellulose biopolymer hydrogel made by DIW 3D printing enabled real‐time alkaline‐nitrogen detection with distinct colorimetric transitions across pH 2–13, suitable for freshness tracking [194]. In another study, a portable micro/nanochannel biosensor achieved rapid, sensitive detection of deoxynivalenol with a detection limit of 1.229 µg/mL [195]. To monitor the heavy metal pollution, 3D‐printed hydrogel platforms were developed for dichromate (Cr_2_O7^2−^) [196] and Hg^2+^ detection (Figure 10b) [197]. Furtherly, a triple‐emission ratio‐metric fluorescence sensor was structured with deep learning assisted processing, enhancing the signal‐to‐noise ratio [198]. The integration of deep learning algorithms not only improves interference resistance but also facilitates monitoring speed by optimizing sensor response diversity, demonstrating a promising strategy for advancing real‐time biosensing applications in challenging condition.

### 5.7 Robotic Manipulation

The hydrogel‐based sensors can be integrated with robotic systems to monitor the motion and operating force in real‐time owing to the conformal adhesion and mechanical compliance. By endowing robots with tactile perception, robotic applications are expanded in delicate object manipulation, particularly for fragile or deformable objects. First, the integration of hydrogel‐based sensors with soft robotic hands has attracted significant interest due to the inherent compatibility to overcome the challenges of precise motion control posed by the nonlinear material properties of soft actuators and the poor integrability of conventional rigid sensors. Li et al. integrated projection lithography fabricated hydrogel strain sensors with pneumatic soft fingers to achieve real‐time bending angle monitoring to improve the motion precision [199]. Additionally, an attachable hydrogel force sensor for grippers enabled in situ force sensing during manipulation; object geometry was identified by analyzing signals from five distributed sensors [200]. Simultaneous measurement of bending angles and operational forces was demonstrated by mounting DIW 3D‐printed hydrogel sensors on a soft robotic hand at an industrial robot end‐effector (Figure 10c), which also supported remote motion control via real‐time feedback [201].

Then, hydrogel‐based sensors can also be used to control joint bionic hand to execute predefined motions. A 3 × 3 hydrogel sensor array capable of controlling bionic hand motion through programmable dot‐matrix coding patterns was introduced [168]. The sensor matrix demonstrated robust performance in robotic hand manipulation even at −40°C. In another design, SLA 3D‐printed hydrogel sensors mounted at the fingertip and finger base captured resistive changes during flexion, offering high sensitivity to complex gesture variations and enabling rapid information transfer for emergency scenarios [110]. Separately, a hydrogel epidermal sensor adhered to skin to acquire EMG signals and actuated synchronized bionic‐hand movements, achieving precise piano‐playing control [202].

Although 3D‐printed hydrogel sensors demonstrate excellent performance in controlled laboratory environments, their translation to real‐world systems is hindered by instability of ionic conductivity under fluctuating humidity, mechanical fatigue at hydrogel‐electrode interfaces, and signal drift caused by dehydration or ion redistribution.

A critical gap in current application‐driven studies is the lack of standardized, accelerated aging protocols evaluating long‐term cyclic stability, adhesion under sweat/oil contamination, and crosstalk in multimodal sensing. Without these metrics, performance comparisons across studies remain largely qualitative. Therefore, the field requires a shift to reliability engineering, where materials, structures, and signal‐processing architectures are codesigned for target use cases.

## 6 Challenges and Perspectives

3D printing technology permits the fabrication of hydrogel‐based sensors with diverse microstructures and customizable geometries, which facilitates the personalized design of flexible sensing devices. However, the following challenges still remain in promoting the widespread adoption of hydrogel‐based flexible sensors across medical, domestic, and industrial applications.

1. The mechanism between 3D printed microarchitectures and the characteristics of hydrogel‐based flexible sensors remains insufficiently characterized. Currently, the absence of quantitative structure–property relationship forces to rely on extensive experimental iterations. This inefficient optimization process not only prolongs development timelines but also results in significant resource expenditure. A fundamental understanding of how specific microstructural parameters govern sensor response characteristics would create more rational design approaches.
2. 3D‐printed hydrogel‐based sensors predominantly exhibit single‐parameter detection capability, significantly limits their practical utility in complex environments where multiple physical detection is required. The development of multidimension flexible sensors capable of concurrent detection of diverse physical signals would substantially expand their applicability across various environmental conditions and operational scenarios.
3. The development of conductive hydrogel‐based flexible sensors faces a fundamental trade‐off between electrical conductivity and mechanical integrity. The electrical insulation of hydrogels necessitates the incorporation of conductive nanofillers to fabricate flexible sensors. Excessive filler loading induces interfacial phase separation that compromises mechanical performance, whereas insufficient loading fails to establish effective conductive pathways. It is necessary to develop advanced conductive nanomaterials with superior hydrogel compatibility, which could simultaneously optimize percolation thresholds and interfacial bonding to achieve balanced electromechanical properties in next‐generation multifunctional sensors.
4. Hydrogel‐based flexible sensors encounter signal processing limitations across biomedical and industrial domains. In human physiological signal and motion monitoring applications, perspiration, and body temperature fluctuations will create signal interference. During industrial implementation, the performance of flexible sensor will be affected by mechanical vibration and environmental particulate‐induced contamination. Machine learning techniques can be introduced to construct sophisticated nonlinear relationships between raw electrical signals and target physical parameters.

3D printed hydrogel sensors demonstrate potential in personalized healthcare monitoring and intelligent human–machine interfaces, with their structural programmability and biocompatibility which establish novel forms for next‐generation wearable devices. The field is developing toward multimaterial integration printing, environmentally adaptive response systems, and wireless communication, while machine learning‐enhanced signal processing promises significant improvements in sensing accuracy and reliability. Future breakthroughs will require interdisciplinary collaboration in functional material development and precision multiscale 3D printing optimization to eliminate the gap between laboratory prototypes and clinical/industrial applications. Particularly promising research directions include creating capsule robot‐integrated platforms for gastrointestinal disease monitoring. Recent progress in wireless capsule endoscopy robots has demonstrated real‐time pH mapping, pressure sensing, and AI‐assisted lesion detection throughout the entire gastrointestinal tract with >95% diagnostic accuracy [203]. Combining these platforms with 3D‐printed MXene‐ or graphene‐based conductive hydrogels would enable multimodal biochemical sensing (pH, glucose, inflammatory markers) and on‐demand drug release triggered by local physiological signals or external magnetic fields, ultimately moving toward closed‐loop “capsule surgeons” for minimally invasive diagnosis and therapy of gastroesophageal reflux disease, inflammatory bowel disease, and gastrointestinal bleeding.

## 7 Conclusion

We summarize the state‐of‐the‐art in 3D printed hydrogel‐based flexible sensors. 3D printing methods applicable to hydrogel sensor fabrication are introduced, and the characteristics of different 3D printing techniques are summarized. Conductive nanomaterials which can impart hydrogel with electrical properties are analyzed. The complex microstructures fabricated directly by 3D printing technologies are introduced, and the capacity of microstructures, such as pores and patterns in improving the sensitivity and range of hydrogel‐based flexible sensors. Furthermore, we overviewed the application of 3D printed hydrogel sensors in human motion detection, physiological signal monitoring, pH sensing, temperature/humidity sensing, glucose/wound monitoring, food safety, and robotic manipulation. However, challenges still remain in optimizing microstructure–property relationships, achieving multidimension sensing, balancing conductivity and mechanical integrity, and enhancing signal processing. Overall, with the development of 3D printing technologies, conductive nanomaterials and hydrogels, miniature, multidimensional and more stable hydrogel‐based sensors will be developed and applied to emerging technology fields.

## Conflicts of Interest

The authors declare no conflicts of interest.
